# Systemic interindividual epigenetic variation in humans is associated with transposable elements and under strong genetic control

**DOI:** 10.1186/s13059-022-02827-3

**Published:** 2023-01-12

**Authors:** Chathura J. Gunasekara, Harry MacKay, C. Anthony Scott, Shaobo Li, Eleonora Laritsky, Maria S. Baker, Sandra L. Grimm, Goo Jun, Yumei Li, Rui Chen, Joseph L. Wiemels, Cristian Coarfa, Robert A. Waterland

**Affiliations:** 1 USDA/ARS Children’s Nutrition Research Center, Department of Pediatrics, Baylor College of Medicine, Houston, TX USA; 2 Keck School of Medicine, University of Southern California, Los Angeles, CA USA; 3 Department of Molecular and Cellular Biology, Baylor College of Medicine, Houston, TX USA; 4 Human Genetics Center, University of Texas Health Science Center at Houston, Houston, TX USA; 5 Department of Molecular & Human Genetics, Baylor College of Medicine, Houston, TX USA; 6 Dan L Duncan Comprehensive Cancer Center, Baylor College of Medicine, Houston, TX USA

**Keywords:** CoRSIV, DNA methylation, DOHaD, Epigenome-wide association study, Genetics, Epigenetic epidemiology

## Abstract

**Background:**

Genetic variants can modulate phenotypic outcomes via epigenetic intermediates, for example at methylation quantitative trait loci (mQTL). We present the first large-scale assessment of mQTL at human genomic regions selected for interindividual variation in CpG methylation, which we call correlated regions of systemic interindividual variation (CoRSIVs). These can be assayed in blood DNA and do not reflect interindividual variation in cellular composition.

**Results:**

We use target-capture bisulfite sequencing to assess DNA methylation at 4086 CoRSIVs in multiple tissues from each of 188 donors in the NIH Gene-Tissue Expression (GTEx) program. At CoRSIVs, DNA methylation in peripheral blood correlates with methylation and gene expression in internal organs. We also discover unprecedented mQTL at these regions. Genetic influences on CoRSIV methylation are extremely strong (median *R*^2^=0.76), cumulatively comprising over 70-fold more human mQTL than detected in the most powerful previous study. Moreover, mQTL beta coefficients at CoRSIVs are highly skewed (i.e., the major allele predicts higher methylation). Both surprising findings are independently validated in a cohort of 47 non-GTEx individuals. Genomic regions flanking CoRSIVs show long-range enrichments for LINE-1 and LTR transposable elements; the skewed beta coefficients may therefore reflect evolutionary selection of genetic variants that promote their methylation and silencing. Analyses of GWAS summary statistics show that mQTL polymorphisms at CoRSIVs are associated with metabolic and other classes of disease.

**Conclusions:**

A focus on systemic interindividual epigenetic variants, clearly enhanced in mQTL content, should likewise benefit studies attempting to link human epigenetic variation to the risk of disease.

**Supplementary Information:**

The online version contains supplementary material available at 10.1186/s13059-022-02827-3.

## Introduction

Genome-wide association studies (GWAS) have revolutionized the field of genetics by identifying genetic variants associated with a range of diseases and phenotypes [1–3]. Nearly 20 years into the GWAS era, however, most human disease risk and phenotypic variation remain unexplained by common genetic variants [2], fueling interest in the possibility that individual epigenetic variation is an important determinant of phenotype [4, 5]. To test this, over the last decade myriad studies have performed genome-scale screens to identify genomic regions at which epigenetic variation is associated with disease. Nearly all these epigenome-wide association studies (EWAS) used commercial arrays manufactured by Illumina (predominantly the HM450 and subsequently the scaled-up EPIC850 array) to assess methylation at CpG dinucleotides (a highly stable epigenetic mark) in peripheral blood DNA [6, 7]. EWAS have uncovered associations between blood DNA methylation and neurological outcomes including Alzheimer’s disease [8], neurodegenerative disorders [9], educational attainment [10], and psychiatric diseases [11]. The HM450 and EPIC arrays were instrumental in discoveries in epigenetic aging [12–14], smoking-induced DNA methylation alterations [15], and understanding how maternal smoking [16] and alcohol consumption [17] affect DNA methylation in newborns. Peripheral blood DNA methylation has been associated with birthweight [18] and body mass index [19].

The Illumina methylation arrays have also played a central role in advancing our understanding of genetic influences on CpG methylation. Genetic variants that correlate with methylation at a specific CpG site (usually in cis) are known as methylation quantitative trait loci (mQTL). Seminal observations of familial clustering of CpG methylation levels [20] led to the first formal study of mQTL [21], which utilized an early version of the Illumina methylation platform. Now, hundreds of studies, nearly all using Illumina methylation arrays, have investigated mQTL in humans [22], enabling estimates of methylation heritability and insights into how genetic effects on disease risk may be mediated by DNA methylation [23] and mechanisms of trans (inter-chromosomal) mQTL effects [24].

Despite these successes, existing and legacy Illumina methylation platforms are not ideal for population epigenetics. The success of GWAS was built upon the HapMap [25] and 1,000 Genomes [26] projects, which systematically mapped out human genome sequence variants so they could be assessed at the population level. So far, however, no “EpiHapMap” project has been conducted. Several large consortium projects, including the Roadmap Epigenome Project [27], the Blueprint Epigenome Project [28], and the International Human Epigenome Consortium [29], focused primarily on characterizing tissue- and cell type-specific epigenetic variation rather than mapping out human genomic regions of interindividual epigenetic variation. The EWAS field therefore relied almost exclusively on Illumina arrays [30] which were designed without consideration of interindividual variation in DNA methylation [31, 32] and generally target CpGs that show little [33–36]. To address this lacuna, we recently conducted an unbiased screen for correlated regions of systemic (i.e., not tissue-specific) interindividual epigenetic variation (CoRSIVs) in the human genome [37]. Because that screen was based on only ten individuals, we set out to assess these regions in a larger cohort to characterize associations among interindividual genetic, epigenetic, and transcriptional variation. In addition to validating CoRSIVs as systemic epigenetic variants, assessing correlations with gene expression, and characterizing associations with transposable elements, we discovered that CoRSIVs exhibit much stronger mQTL than previously observed. Because interindividual variation is essential not just for mQTL detection but also for epigenetic epidemiology, our results have important implications for the EWAS field.

## Results

### Target-capture bisulfite sequencing confirms systemic interindividual variation in DNA methylation

**Fig. 1:**
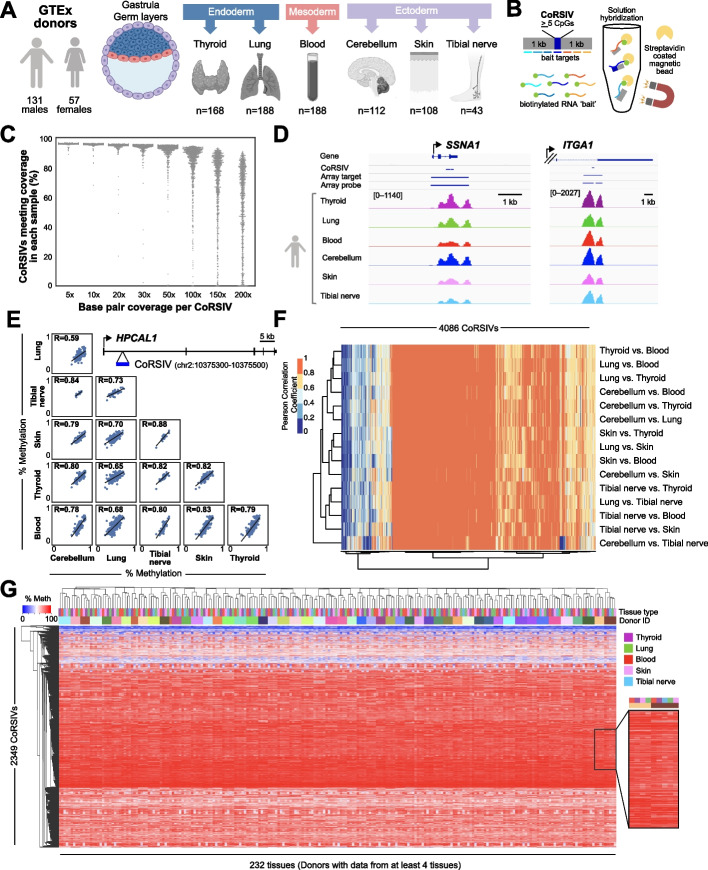
Target-capture bisulfite sequencing in 807 GTEx samples confirms systemic interindividual epigenetic variation at CoRSIVs. **A** DNA samples were obtained from multiple tissues (representing the three embryonic germ layers) from each of 188 GTEx donors. **B** CoRSIV capture process using Agilent reagents. **C** Percentage of CoRSIVs for which target-capture bisulfite sequencing achieved various read depths; each point represents one of 807 samples. **D** Plots of read depth at two target regions illustrate specificity of targeting across all six tissues. The *Y*-axis scales are same for each region and indicated for thyroid. **E** Scatter plots between all possible tissue pairs illustrate high inter-tissue correlations at a CoRSIV within *HPCAL1*. **F** Heat map of inter-tissue correlations across 4086 CoRSIVs shows generally high correlation coefficients between all possible tissue pairs. **G** For the 232 tissue samples from 53 donors with data on at least 4 tissues (excluding cerebellum), unsupervised hierarchical clustering of methylation data at 2349 fully informative CoRSIVs groups perfectly by donor

In collaboration with the NIH Genotype-Tissue Expression (GTEx) program [38], we conducted target-capture bisulfite sequencing to quantify DNA methylation at 4641 gene-associated CoRSIVs in multiple tissues representing the three embryonic germ layers from each of 188 GTEx donors (807 samples total) (Fig. 1A, B). For donor and sample information and regions targeted, see Additional file 2: Table S1 and S2, respectively. The raw data have been deposited in a controlled-access public repository (dbGaP accession phs001746.v2.p1) linked to GTEx identifiers. We achieved high capture efficiency (Additional file 1: Fig. S1A, B, C); over 90% of targeted regions were covered at 30x sequencing depth in nearly all 807 samples (Fig. 1C, D, Additional file 1: Fig. S1B). Data on read counts, alignment efficiency, bisulfite conversion efficiency, and duplication rate are provided (Additional file 2: Table S3). A small subset of difficult-to-capture regions failed to meet coverage criteria in all libraries (Additional file 1: Fig. S1C, Additional file 2: Table S4). A set of Y-chromosome regions included in the capture enabled us to confirm that all 807 samples are of the correct sex (Additional file 1: Fig. S1D), indicating reliable sample handling.

CoRSIVs were identified based on unbiased genome-wide assessment of DNA methylation in thyroid, heart, and brain [37]. Our first goal, therefore, was to examine additional tissues to confirm systemic interindividual variation (SIV) at these regions. High inter-tissue correlation in DNA methylation is the hallmark of SIV (Fig. 1E). Of the 4641 genic CoRSIVs targeted, the 4086 that satisfied coverage criteria in at least 10 donors in every possible pair of tissues were evaluated. Most of these showed high positive inter-tissue correlations (Pearson *R*>0.6) across all possible tissue pairs (Fig. 1F, Additional file 1: Fig. S1E, Additional file 2: Table S5), confirming SIV. Accordingly, unsupervised clustering of methylation data at the 2349 CoRSIVs covered in all 5 tissues (except cerebellum) across 53 donors grouped perfectly by the donor (Fig. 1G, Additional file 2: Table S6). This clustering was not associated with sample-level variation in capture efficiency (Additional file 2: Table S7). As DNA methylation in the cerebellum often differs from that in other brain regions [39], including cerebellum in this analysis resulted in a minor cerebellum cluster (Additional file 1*:* Fig. S1F); nonetheless, high inter-tissue correlations were maintained (Additional file 1*:* Fig. S1G). Of greatest relevance to epigenetic epidemiology, CoRSIV-specific scatter plots of methylation in brain, thyroid, skin, lung, and nerve versus that in blood show that methylation in blood generally serves as a proxy for methylation in other tissues (five tissues vs. blood). By comparison, in an HM450 study of 122 individuals [39], correlations between methylation in 4 brain regions vs. blood averaged only 0.2 and rarely exceeded 0.5. Although the inter-tissue scatter plots at CoRSIVs commonly show either a uniform distribution or three clusters (suggesting a single-genotype effect) (Additional file 1*:* Fig. S2), other patterns observed include 2, 4, and 5 distinct clusters (Additional file 1: Fig. S3). Consistent with our earlier study [37], in all six tissues almost every CoRSIV displayed an interindividual methylation range >20% (median range 40–42%) (Additional file 1: Fig. S4). Together, these results validate these CoRSIVs as systemic individual variants, essentially epigenetic polymorphisms.

### Gene expression in internal organs correlates with CoRSIV methylation in blood

Compared to genetic epidemiology, epigenetic epidemiology is complicated by the inherent tissue-specificity of epigenetic regulation [5]. Because nearly all EWAS are based on measuring methylation in peripheral blood DNA, attempts to discover associations with, for example, Alzheimer’s disease [9] or schizophrenia [40] are implicitly predicated on the assumption that methylation variants in blood associate with epigenetic regulation in the brain. Of those on the Illumina arrays, however, such probes are the exception [39, 41]. We therefore used our target capture bisulfite sequencing data and transcriptional profiling (RNA-seq) data from GTEx to test for cross-tissue correlations between CoRSIV methylation and expression of associated genes.

Of 3768 CoRSIV-associated genes, over half showed appreciable expression in at least 5 of the six tissues under consideration (Additional file 1: Fig. S5A, B). Tibial nerve was excluded from this analysis due to low sample size; for each other tissue, both CoRSIV methylation and gene expression data were available for at least 60 individuals (Additional file 1: Fig. S5C). Tissues that are difficult to sample non-invasively (thyroid, lung, and cerebellum) were considered “target” tissues. Within each of these, we identified all CoRSIV-gene pairs for which gene expression is associated with CoRSIV methylation (FDR<0.05) (Additional file 1: Fig. S6A, B show two examples). Relative to those within a gene body, CoRSIVs located within 3 kb of either the 5′ or 3′ end of a gene showed predominantly negative correlations between methylation and gene expression (OR=2.84, *P* = 0.002) (Additional file 1*:* Fig. S6C).

For each CoRSIV-gene pair showing an expression vs. methylation association in a target tissue, we next asked whether methylation measured in easily accessible “surrogate” tissues (blood or skin) is associated with expression in the target tissue. Of 156 genes for which expression was correlated with CoRSIV methylation in the thyroid, for example, 122 (75%) showed a significant correlation and in the same direction when methylation in blood was used as the independent variable (Additional file 1: Fig. S6D). Likewise, in the lung and cerebellum, at least 75% of all methylation-expression correlations were detected when methylation in blood was used to infer expression (Additional file 1: Fig. S6D). In the other surrogate tissue, skin, this figure was slightly lower (60%). These data demonstrate that, at gene-associated CoRSIVs, methylation measurements in easily accessible tissues like blood can be used to draw inferences about epigenetic regulation in internal organs, a major advantage for epigenetic epidemiology.

### Genetic influences on methylation at genic CoRSIVs are strong and biased

The Genetics of DNA Methylation Consortium (GoDMC) recently analyzed HM450 and genotyping data on nearly 33,000 people in 36 cohorts [42] and documented mostly modest effects; for 75% of the *cis* mQTL associations, the genetic variant explained less than 5% of the variance in methylation. In the largest unbiased study of human mQTL, Busche et al. [43] performed whole-genome bisulfite sequencing in 43 female twins and concluded that environment, not genetics, is the main source of interindividual variation in DNA methylation.

**Fig. 2:**
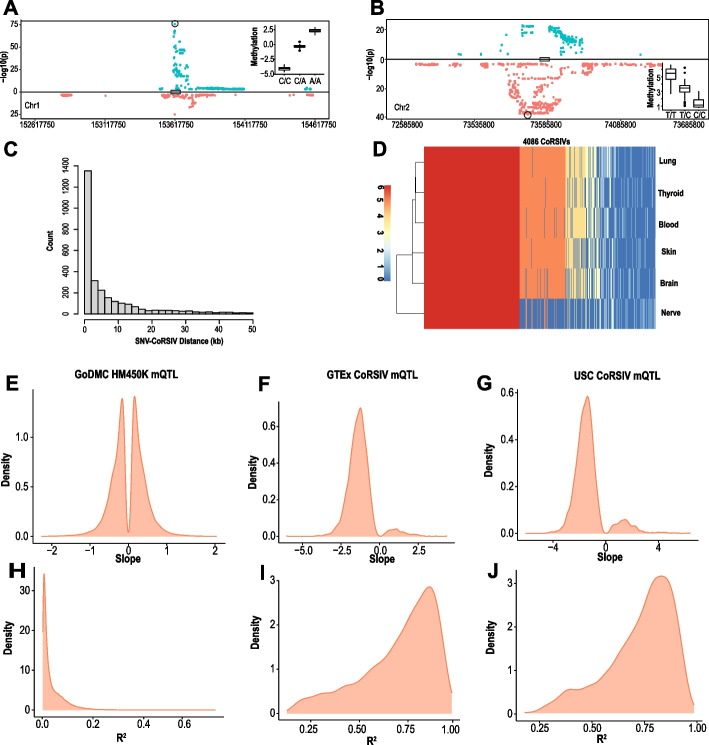
Genetic influences on CoRSIV methylation are strong and biased. **A**, **B** Representative plots of mQTL associations at individual CoRSIVs on chromosomes 1 and 2, respectively. Significant associations are shown for all SNVs within 1Mb of each CoRSIV; positive and negative beta coefficients are plotted in blue and red, respectively. The most significant SNV (Simes SNV) is circled. Insets show average CoRSIV methylation vs. Simes SNV genotype. **C** Distribution of distances between CoRSIVs and corresponding Simes SNVs. **D** For each of 4086 CoRSIVs, heat map depicts the number of tissues in which the Simes SNV falls within the same haplotype block, illustrating the largely systemic nature of mQTL at CoRSIVs. **E** Distribution of beta coefficients of significant Simes mQTL associations for the GoDMC blood mQTL data [42]. **F** Distribution of beta coefficients of significant Simes mQTL associations at 3723 CoRSIVs in blood DNA from 188 GTEx donors. **G** Distribution of beta coefficients of significant Simes mQTL associations across 2939 CoRSIVs in blood DNA from 47 newborns (USC). **H** Distribution of Simes mQTL *R*^2^ (goodness of fit) for the GoDMC data. **I** Distribution of Simes mQTL *R*^2^ at CoRSIVs (GTEx, blood). **J** Distribution of Simes mQTL R^2^ at CoRSIVs (USC samples)

We wondered to what extent individual variation in CoRSIV methylation is explained by genetic variation in *cis*. Within each CoRSIV, methylation of multiple CpGs is highly correlated [37]; we therefore tested for genetic associations with average CoRSIV methylation, rather than at the CpG level. Also, given the multiplicity of mQTL associations at each CoRSIV (median 22 SNVs with *P*<10^−10^ per CoRSIV, Additional file 1: Fig. S7), rather than attempt to detect all possible SNV-CoRSIV associations, we employed the Simes correction [44] to identify the single SNV most strongly associated with methylation at each CoRSIV (lowest *p* value, adjusted for multiple testing) (Fig. 2A, B, Additional file 1: Fig. S8, Additional file 2: Table S8; listed *p* values are adjusted for multiple testing). This approach conservatively tests each CoRSIV for evidence of genetic influence on its methylation and is much more powerful than those we were able to employ in our earlier study [37] based on just 10 individuals.

Although we tested all SNVs within 1 Mb, “Simes SNVs” were generally proximal to the CoRSIV, 72% within 10 kb (Fig. 2C, Additional file 1: Fig. S9). Remarkably, although the Simes procedure was carried out independently in each tissue, at each CoRSIV the exact same SNV in many cases yielded the strongest mQTL association in all or most of the tissues (Additional file 1: Fig. S10A, B). When we asked how often the Simes SNV was within the same haplotype block in all or most tissues, concordance was even stronger (Fig. 2D), indicating the systemic nature of genetic influences on methylation at genic CoRSIVs.

Previous studies of mQTL using the HM450 array [22, 42] consistently report beta coefficients balanced on both sides of zero, as we found by employing the Simes procedure to the GoDMC data (Fig. 2E). Conversely, most *cis* mQTL associations at genic CoRSIVs show a negative beta coefficient (i.e., the major allele is associated with higher methylation) (Fig. 2F). This imbalance held not just for Simes SNVs, but for all mQTL SNVs (Additional file 1: Fig. S11). The strength of mQTL associations at genic CoRSIVs also appears to be without precedent [22, 42]. In the GoDMC data, for example, few Simes mQTL associations show an *R*^2^ > 0.2 (Fig. 2H); at CoRSIVs, the median *R*^2^ = 0.76 (Fig. 2I, Additional file 1: Fig. S12). This tendency for high-*R*^2^ mQTL was largely independent of the distance between CoRSIV and SNV (Additional file 1: Fig. S13).

We made several attempts to disprove these surprising findings. Though unlikely (because each CoRSIV contains at least 5 CpGs [37]), we first asked whether the strong mQTL effects could be caused by SNVs abrogating CpG sites within CoRSIVs. Of SNVs present in our sample of 188 individuals, at least one did overlap a CpG within most of the CoRSIVs we surveyed. The distributions of beta coefficient and *R*^2^ values of Simes mQTL associations for the 1155 CoRSIVs without any such overlaps, however, were nearly identical to those of the 2759 with SNV-CpG overlaps (Additional file 1: Fig. S14). We next asked whether, instead of affecting CpG sites, SNVs within CoRSIVs might introduce an artifact by compromising the binding of the baits used for target capture. Despite their small size (median 200 bp), most CoRSIVs contain 2 or more SNVs (Additional file 1: Fig. S15A); however, neither the beta coefficients nor the *R*^2^ values of the Simes mQTL associations were strongly associated with the number of SNVs per CoRSIV (Additional file 1: Fig. S15B, C). Together, these data indicate that the strong and biased mQTL effects we detected are not due to SNVs within CoRSIVs.

For a complementary analysis, we employed a haplotype-based approach to assess genetic influences on CoRSIV methylation. We used phased genotype data from GTEx to infer each individual’s haplotype within the haplotype block overlapping each CoRSIV and assessed correlations between CoRSIV methylation and haplotype allele sum (sum of minor alleles in each individual) (Additional file 1: Fig. S16A). This analysis yielded a preponderance of negative coefficients, and local haplotype explained much of the variance in methylation (median *R*^2^ = 0.43) (Additional file 1: Fig. S16B, Additional file 2: Table S9), consistent with the mQTL analysis.

Lastly, to independently validate genetic effects on CoRSIV methylation, we performed CoRSIV-capture bisulfite-sequencing and SNV genotyping in 47 individuals from a different (non-GTEx) population (USC cohort). To ensure computational independence, a separate member of our laboratory wrote new code for the Simes mQTL analysis. The USC results corroborated the negative bias and high *R*^2^ of mQTL effects at CoRSIVs (Fig. 2G, J, Additional file 2: Table S10). An independently performed haplotype-based analysis likewise corroborated the results obtained on the GTEx samples (Additional file 1: Fig. S16C, Additional file 2: Table S11). Together, these additional analyses and data indicate that the strong and biased genetic influences on methylation at CoRSIVs are genuine.

We wondered how the total amount of mQTL we detected at genic CoRSIVs compares with that reported by the GoDMC [42], which used HM450 arrays to study 33,000 people. With 3 genotype calls possible at each SNV, the average methylation difference (delta) associated with each SNV can be calculated from the mQTL beta coefficient (Additional file 1: Fig. S17A). And, since the mQTL *R*^2^ measures what proportion of this delta is explained by SNV genotype, the product (delta)x(*R*^2^) measures the absolute methylation variation explained by SNV genotype. To make our results interpretable, we initially assessed (delta)x(*R*^2^) based on beta values (rather than using the M-value transformation). Across all CoRSIV mQTLs (*P* < 10^−10^), median (delta)x(*R*^2^) was 24.6% methylation (Additional file 1: Fig. S17B); for a CoRSIV with an *R*^2^ near the median (0.76), this equates to an interindividual range of 32.4% methylation, within the normal range for CoRSIVs (Additional file 1: Fig. S4). To compare our results with those of GoDMC [42], whose coefficients were provided based on *M* values, we repeated our analysis after applying the *M* value transformation. At the CoRSIVs we assayed, the total methylation variance explained by genetics (sum of (delta)x(*R*^2^)) was 72-fold greater than that detected by GoDMC [42] (Additional file 1: Fig. S17C, D, E), the largest study of human mQTL ever reported.

Genetic influences on tissue-specific expression (eQTL) can be mediated by mQTL [23, 45]. Given the strong mQTL effects at genic CoRSIVs, we used data from GTEx [46] to ask whether Simes SNVs are enriched for eQTL. Consistent with the analysis of GTEx data overall [46], many eQTL effects were shared among non-brain tissues, whereas eQTL associations in the brain and blood were more distinct (Additional file 1: Fig. S18A). Relative to all common variants, which have a 50% chance of being associated with expression of a nearby gene [46], a bootstrapping analysis indicated that Simes SNVs are 3.4-fold more likely to show eQTL effects (Additional file 1: Fig. S18B). The distributions of magnitude, slope, and SNV-eGene distance for eQTL effects at Simes SNVs were similar to those of all common variants (Additional file 1: Fig. S18C, D). Future studies will be required to determine if the enriched eQTL at Simes SNVs is in some cases mediated by CoRSIV mQTL.

### CoRSIVs occur in genomic regions with far-reaching enrichments in transposable elements

The earliest known examples of systemic interindividual epigenetic variants in mammals are mouse metastable epialleles such as *agouti viable yellow* and *axin fused*, both of which resulted from retrotransposition of an intracisternal-A particle (an LTR-retrotransposon) [47, 48]. We previously showed that CoRSIVs are enriched for direct overlaps with LINE, SINE, and ERV retrotransposons [37]; we provide a more granular analysis of those overlaps here (Additional file 1: Fig. S19). Given the ability of transposable elements for long-range regulation of transcriptional and epigenetic dynamics in the early embryo [49, 50], we asked whether the exceptional behavior of CoRSIVs might be associated with specific classes of repetitive elements working over long genomic distances.

**Fig. 3:**
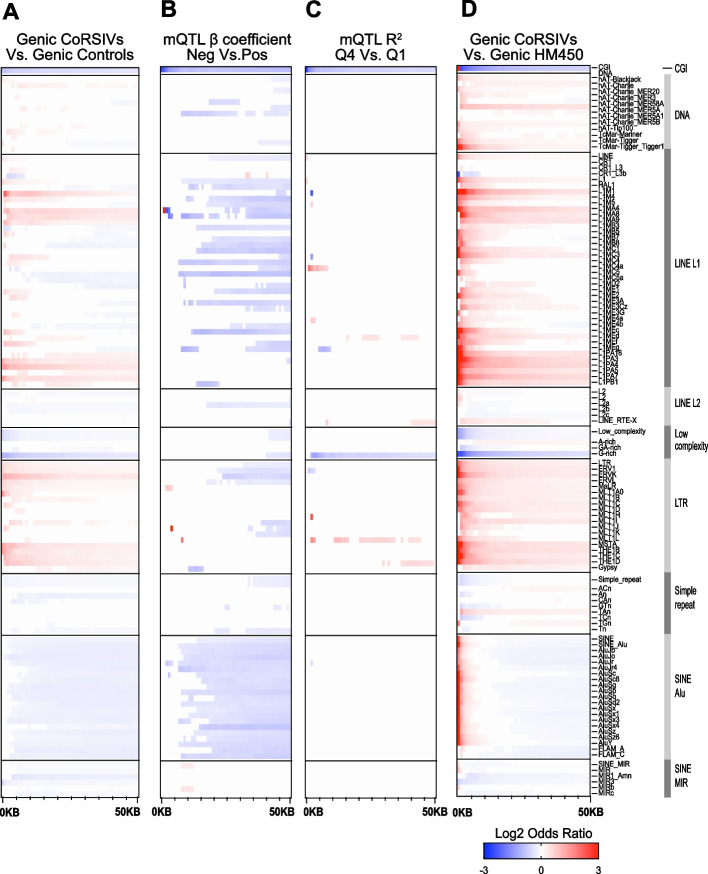
Genic CoRSIV-flanking regions show long-range enrichments and depletions for specific classes of transposable elements. **A** Using 1 kb step sizes, each plot shows significant enrichments or depletions for CpG islands (CGI) and subclasses within each of 8 classes of transposable element within 50 kb of genic CoRSIVs. Compared to control regions, CoRSIV-flanking regions show long-range depletion of CpG islands and enrichment of specific classes of LINEs and LTRs. **B** Compared to CoRSIVs showing a positive mQTL beta coefficient, those with negative coefficients are depleted for CpG islands and show long-range depletion of specific LINE1s and all subclasses of Alus. **C** The strength of mQTL associations at CoRSIVs (R^2^ in 4^th^ vs. 1^st^ quartile) is not associated with widespread differences in genomic content of transposable elements. **D** Compared to regions in which HM450 probes are located, CoRSIVs show short- and long-range enrichments for many subclasses of LINE1 and LTR retrotransposons

Relative to a set of control regions matched to genic CoRSIVs by chromosome, size, and CpG density [37], in regions flanking genic CoRSIVs we detected long-range depletion of CpG islands and enrichments of specific classes of LINE and LTR retrotransposons (Fig. 3A, Additional file 2: Table S12). Similar and stronger enrichments were detected in comparison with size-matched tissue-differentially methylated regions (tDMRs) [37] (Additional file 1: Fig. S20). Interestingly, enrichments relative to control regions (Fig. 3A) were strongest among the evolutionarily youngest subclasses, the LINE1-PA elements [51] among LINEs, and ERV-K elements [50] among LTRs.

We next asked whether either the negative bias (i.e., the major allele associating with higher methylation) or the strength of mQTL associations at CoRSIVs might be associated with transposable elements in flanking genomic regions. Compared to genic CoRSIVs showing a positive mQTL beta coefficient, those characterized by negative coefficients were depleted for CpG islands (Fig. 3B). There were no robust short-range associations of transposable elements with “negative mQTL” CoRSIVs; rather, at distances > 5–10kb from the origin, they show extensive long-range depletion of specific LINE1 and all classes of Alu elements (Fig. 3B, Additional file 2: Table S13). Surprisingly, the strength of mQTL at genic CoRSIVs was not associated with widespread differences in genomic content of transposable elements. Relative to those in the bottom quartile for *R*^2^, mQTL effects in the top quartile showed proximal and long-range depletion in just CpG islands and G-rich low-complexity repeats (Fig. 3C, Additional file 2: Table S14).

As most human mQTL data are based on the HM450 array, we next compared genomic regions flanking genic CoRSIVs with those flanking genic HM450 probes, finding striking differences. Although the HM450 array specifically targets CpG islands, these are more strongly enriched within 1 kb of genic CoRSIVs (Fig. 3D, Additional file 2: Table S15); at greater distances, CoRSIV-flanking regions are relatively depleted of CpG islands. Compared to genomic regions containing genic HM450 probes, those housing genic CoRSIVs show strong short-range (1–2 kb) enrichments in LINE1, LTR, and Alu elements (Fig. 3D). The LINE1 and LTR enrichments gradually weaken but extend to at least 50 kb from the origin. Enrichments for Alu extend only to ~5 kb; at greater distances, regions flanking genic CoRSIVs are relatively depleted (Fig. 3D). These enrichments were not unique to genic CoRSIVs; the full set of 9926 CoRSIVs showed similar patterns of enrichment relative to matched control regions, tDMRs, and HM450 probes (Additional file 1: Fig. S21). These observations suggest a straightforward explanation for the strong and biased mQTL effects at CoRSIVs. To limit hybridization artifacts, the Illumina methylation arrays avoided genomic regions rich in transposable elements. But these are the same regions in which SIV tends to occur. Given the potentially deleterious consequences of transcriptional activation of retrotransposons, the strong and negative mQTL beta coefficients at CoRSIVs could reflect evolutionary selection for genetic variants favoring their methylation and silencing. In support of this, values of Tajima’s D (a test statistic assessing evidence of evolutionary selection) are higher in CoRSIVs compared to control, tDMR, or HM450 probe regions (Additional file 1: Fig. S22, Additional file 2: Table S16).

### CoRSIV flanking regions are enriched for heritability of disease

**Fig. 4:**
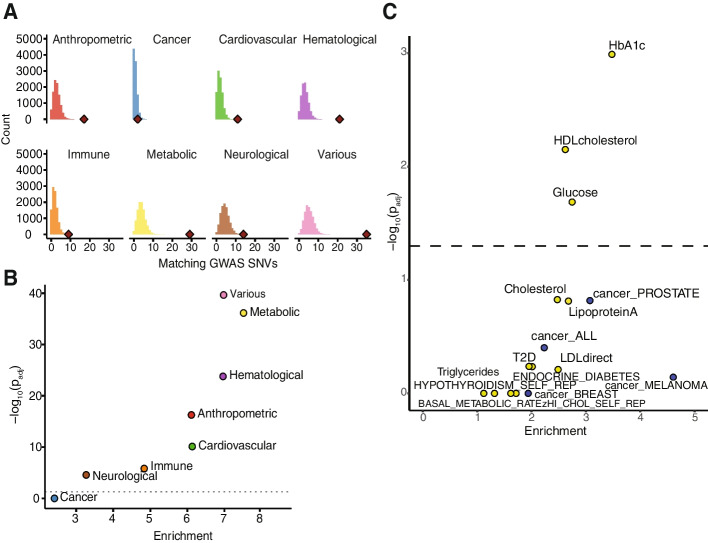
CoRSIV mQTL SNVs are enriched for GWAS associations. **A** Within each of 8 disease/phenotype categories, the histogram shows the null distribution obtained by permutation testing for overlap of GWAS SNVs with SNVs randomly sampled within 1Mb of each CoRSIV. The red diamond shows the actual number of overlaps between CoRSIV mQTL SNVs and GWAS SNVs. Numbers of GWAS SNVs considered in each category are anthropometric: 8106, cancer: 3163, cardiovascular: 4816, hematological: 7461, immune: 5263, metabolic: 10,121, neurological: 14,741, and various: 14,573. **B** Statistical significance (Bonferroni-adjusted *p*-value) vs. fold enrichments for the analysis in **A**. Strong and statistically significant enrichments were found for all outcomes except cancer. **C** Statistical significance (Bonferroni-adjusted *p*-value) vs. fold enrichments for 8 metabolic traits and 4 cancer outcomes from the LDSC analysis confirms that the vicinity of CoRSIVs is enriched for heritability of metabolic traits

Across diverse outcomes including Alzheimer’s [23], chronic obstructive pulmonary disease [52], obsessive-compulsive disorder [53], and cardiovascular disease [54], integrative analyses of GWAS and DNA methylation profiling data increasingly indicate that mQTL mediates associations between genetic variation and risk of disease. We therefore asked whether the strong mQTL effects identified at genic CoRSIVs are associated with genetic variants identified by GWAS. Indeed, permutation testing indicates that SNVs identified in our mQTL analysis are enriched for SNVs implicated in metabolic, hematological, anthropometric, cardiovascular, immune, neurological, and various other diseases (Fig. 4A, B, Additional file 2: Table S17). By contrast, despite an abundance of CoRSIV-associated genes linked to cancer [37], no enrichment was found relative to cancer GWAS SNVs (Fig. 4A, B). Notably, a recent HM450 analysis employing these same categories [24] found nearly opposite categorical enrichments with *trans*-mQTL loci. With the caveat that 90% of GWAS alleles impact multiple traits [55], it is interesting that cancer traits are not enriched. This may indicate that CoRSIV methylation plays no role in this maladaptive phenotype, or reflect dilution of effects across multiple cancer subtypes and various genetic pathways leading to cancer [56]. Overall, and particularly considering that Simes SNVs are enriched for eQTL, these results are consistent with the possibility that human genetic variants influence disease risk via mQTL effects at CoRSIVs.

As a complementary analysis, we used LD score regression (LDSC) [57] to determine if, in the vicinity of genic CoRSIVs, there is enrichment for heritability of metabolic phenotypes and cancer. GWAS summary statistics from the UK Biobank representing 12 metabolic traits and 4 cancer outcomes were downloaded [58]. As nearly all Simes SNVs are within 20 kb of their associated CoRSIV (Fig. 2C), we evaluated genomic regions encompassing genic CoRSIVs ± 20 kb. Consistent with our results based on direct overlap with Simes SNVs, individual LDSC models focused on each outcome detected significant enrichment for 3 metabolic outcomes (HbA1c, HDL cholesterol, and glucose) but none for cancer (Fig. 4C). As suggested by Finucane et al. [57], we repeated these analyses including in each a full “baseline” model comprising 53 sequence and epigenomic features. Enrichment for heritability of two of the metabolic traits, HbA1c and HDL cholesterol, was attenuated but remained significant (Additional file 1: Fig. S23A). The baseline-adjusted analysis (Additional file 1: Fig. S23B) confirmed strong evolutionary conservation in the vicinity of genic CoRSIVs. Also, significant enrichments for coding regions and transcription start sites may explain the attenuated associations with metabolic outcomes. Regardless, we would argue that because CoRSIVs were identified based solely on SIV in DNA methylation, it is inappropriate to penalize them for association with genic and regulatory features. Hence, the LDSC results corroborate that CoRSIV-flanking regions are enriched for heritability of metabolic disease.

## Discussion

Following up on our previous screen for human CoRSIVs [37], here we have, for the first time, demonstrated the feasibility of studying these regions at the population level using target-capture bisulfite sequencing. Performing these analyses on donors from GTEx allowed us to integrate our methylation data with genome sequence and gene expression data on these same individuals. As expected, our results validated SIV at the CoRSIVs we analyzed and indicate the ability to use methylation profiling in peripheral blood to draw inferences about epigenetic regulation in various organs of the body. More surprisingly, our analyses of genetic influences on CoRSIV methylation indicate an unprecedented level of mQTL at these regions. Also unlike previous reports, our mQTL analysis showed strongly biased beta coefficients (i.e., the major allele associated with higher methylation). Lastly, we found evidence that genomic regions encompassing CoRSIVs are enriched for the heritability of human disease traits.

Though unprecedented, the extremely strong mQTL effects at the CoRSIVs we surveyed are unsurprising. Because variation at each SNV is fixed (ranging from 0 to 2 copies of the minor allele), the best way to increase the power of mQTL detection is to focus on CpG sites with the greatest interindividual range of DNA methylation. Other than our work [37, 59, 60], we are not aware of previous studies that took this approach. Instead, nearly all investigations of human mQTL have employed Illumina arrays [22], which do not target interindividual variants. One may question the validity of quantitatively comparing our mQTL results with those of GoDMC [42]. After all, GoDMC analyzed HM450 data on 420,000 CpG sites across nearly 33,000 individuals, whereas we analyzed target-capture bisulfite sequencing data on 4086 CoRSIVs in just 188 individuals. But although the targeted regions and studied populations differ, both analyses employed the same statistical method for mQTL detection. Because GoDMC performed their mQTL analyses using *M* values (a transformation of the Beta value intended to improve normality), we also transformed our percent methylation data to *M* values for this comparison. Therefore, despite the different approaches and vastly dissimilar numbers of subjects considered, our analysis is quantitatively comparable to that of Min et al. [42]. Our ability to detect more mQTL than ever before despite surveying a much smaller number of CpG sites speaks to the importance of targeting the right CpGs. Known human CoRSIVs comprise just 0.1% of the genome; whilst some may question the wisdom of focusing on such a small fraction of genomic CpG sites, common human sequence variants comprise only ~0.3% of the genome [26] but have been a major focus of the GWAS field for the last 20 years.

In addition to the extremely strong mQTL effects at genic CoRSIVs, we are not aware of previous studies showing a bias in mQTL regression coefficients (Fig. 2F, G). The mQTL bias at genic CoRSIVs reflects that the major allele is generally associated with higher methylation. This is consistent with the enrichment of L1 and LTR transposable elements in the vicinity of CoRSIVs (Fig. 3), because these tend to locate in heterochromatic regions [61]. During human pre-implantation development, when methylation at CoRSIVs is thought to be established [37, 62], widespread genomic de-methylation leads to transient transcriptional activation of transposable elements, prior to their re-methylation and silencing in differentiated tissues [63]. The high density of L1 and LTR retrotransposons in CoRSIV-flanking regions therefore raises the question of whether mQTL effects at CoRSIVs reflect modulation of the *establishment* of de novo or early embryonic *maintenance* of existing zygotic methylation. In this regard, it is striking that, in mice, L1 elements and IAPs (a class of LTR retrotransposons) are preferentially methylated in sperm and not oocytes, whereas Alus show the opposite pattern (methylated in oocytes but not in sperm) [64]. These observations mirror our data on transposable element enrichments in regions flanking CoRSIVs (Fig. 3A). The biased mQTL beta coefficients at CoRSIVs lead us to speculate that they could reflect evolutionary selection for genetic variants that maintain methylation marks in the paternal genome, potentiating transgenerational epigenetic inheritance as observed at the murine metastable epiallele *axin fused* [65].

As DNA methylation can act as an intermediary molecular mechanism linking genetic variation to tissue-specific transcriptional regulation [23, 45], mQTLs may provide mechanistic insights into how genetic variants influence gene expression. In this regard, the dramatically different nature of mQTL effects at genic CoRSIVs, in terms of both strength and allelic bias, indicates that we have uncovered a fundamentally different component of epigenetic regulation compared to CpGs represented on the HM450 and EPIC arrays which have largely been the focus of the field [22]. Also, our observation that SNVs wielding the strongest mQTL effects at genic CoRSIVs are enriched for eQTL suggests a mechanistic pathway in which genetic effects on CoRSIV methylation modulate tissue-specific gene expression. On the other hand, 16% of CoRSIVs showed weak effects explaining less than half of the interindividual variation (Fig. 2I). These are candidate metastable epialleles. Future large human studies can better characterize genetic effects on CoRSIV methylation and elucidate true epipolymorphisms (i.e., metastable epialleles) at which a majority of interindividual epigenetic variation is unexplained by genetics, such as the non-coding RNA *nc886* (also known as *VTRNA2-1*) [17, 66]. Combining data on such regions with those on recently identified murine metastable epialleles [67] may enable comparative genomic approaches to characterize sequence features that confer epigenetic metastability, informing in silico identification of metastable epialleles in other mammalian species.

Many important questions remain unanswered by our study. Our initial identification of CoRSIVs was based on ten White, non-Hispanic individuals. Reflecting the GTEx study overall, 90% of the donors included in this current study are also White, non-Hispanic. Although our previous studies [37, 59, 60] indicate that SIV regions identified in White, non-Hispanics generally also show SIV in other ancestry groups, future studies screening for SIV directly in other populations may identify CoRSIVs specific to those ancestry groups. Also consistent with the GTEx study population overall, most donors studied here were between 50 and 70 years old (Additional file 2: Table S1). Considering the influence of age on epigenetic marks [12], one might ask to what extent interindividual variation at CoRSIVs is influenced by age. Notably, the validation studies we performed to corroborate mQTL effects at CoRSIVs (Fig. 2G, J) were based on peripheral blood of newborns yet showed nearly identical profiles of mQTL slope and variance explained, arguing that age is not a major factor in the regulation of systemic interindividual epigenetic variation. Compared to our initial screen which surveyed thyroid, heart, and cerebellum, here we evaluated SIV in 4 additional tissues, with at least one representing each germ layer lineage (Fig. 1A). Hence, whereas our results confirm high inter-tissue correlation coefficients across most tissue pairs for ~90% of genic CoRSIVs (Fig. 1F), many more tissues and cell types remain to be evaluated. The small fraction of genic CoRSIVs with low inter-tissue correlations (Fig. 1F) may reflect false positives in our original screen, or possibly exhibit interindividual variation across specific tissue lineages not evaluated here.

The generally strong mQTL at CoRSIVs is not necessarily due to the systemic nature of their interindividual variation. Most of these same regions would have been detected if, instead of our original three-tissue screen [37], we had conducted an unbiased genome-wide screen for interindividual variation in, say, peripheral blood leukocytes. In addition to CoRSIVs, such an experiment would detect interindividual variants specific to blood. Rather than interindividual variation intrinsic to leukocytes, however, many of these reflect interindividual variation in leukocyte composition (ratio of B cells to T cells, for example) [68]. We would argue that such variants are not *bona fide* interindividual epigenetic variants. Because most human tissues exhibit such cellular heterogeneity, the specific composition of which can differ among individuals and disease states, interindividual variation observed in just one tissue is difficult to interpret. CoRSIVs, on the other hand, are unaffected by individual differences in tissue cellular composition [37]; like sequence variants, they are stable epigenetic variants intrinsic to essentially all cells in an individual. The CpG methylation profile at CoRSIVs can therefore reasonably be considered a readout of an individual’s epigenome, enabling adoption of concepts and applications developed for genomics, such as GWAS. Given the strong influence of genetics on methylation at CoRSIVs, one might ask whether profiling CoRSIV methylation offers additional information beyond that obtained by genotyping. We anticipate many advantages. First, as multiple genetic variants influence methylation at each CoRSIV (Additional file 1: Fig. S7), CoRSIV methylation can be viewed as an integrative readout of these influences. Also, GWAS variants may logically be prioritized based on known mQTL effects at CoRSIVs, just as investigators now prioritize GWAS hits based on evidence of eQTL [69]. In fact, mQTL effects at CoRSIVs may in some cases mediate eQTL. Lastly, whereas our current data on CoRSIV mQTL is based on a mostly White, non-Hispanic cohort in the USA, it is possible that additional sources of variation (for example, due to periconceptional environment [37, 59, 60]) will be uncovered as CoRSIVs are studied in a broader range of ancestral and cultural contexts, providing insights into gene by environment interactions.

## Conclusions

For over 10 years, the Illumina methylation platform has been the predominant tool for population studies of DNA methylation [22, 30]. A major reason is that it interrogates a stable subset of CpG sites within the human genome (yielding one quantitative value for each), simplifying data sharing and integration across multiple studies and populations. Nonetheless, the platform has a major and undeniable shortcoming in the context of population epigenetics: most CpGs included do not show appreciable interindividual variation [33–36]. Here we have shown that focusing on systemic methylation variants enables the identification of far stronger mQTL than has been detected by the Illumina arrays [42]. We anticipate that the greater population variance at CoRSIVs will also improve the power of studies aiming to associate epigenetic variation with risk of disease. Generating the data to explore associations between CoRSIV methylation and a wide range of human diseases is beyond the scope of this study. However, though grossly underrepresented on the HM450 and EPIC arrays (less than 1% of EPIC probes overlap known SIV regions; see annotated list in Additional file 2: Table S18), CoRSIVs are often among the top “hits” in existing EWAS [70]. Indeed, these stable [36, 60, 71], systemic epigenetic variants are already showing great promise for disease prediction [72–78]. We suggest that improving the coverage of CoRSIVs would enhance the utility of the Illumina EPIC array for the study of population epigenetics. Additionally, we wish to make our validated human CoRSIV-capture reagents available to the field to facilitate the study of these systemic variants. The list of known human CoRSIVs is currently incomplete, and screening is underway to identify more, including in various ancestry groups.

## Materials and methods

### Study samples

We obtained de-identified genomic DNA from multiple tissues of 188 donors in collaboration with NIH Genotype-Tissue Expression (GTEx) program [38] (total of 807 samples). Informed consent was obtained by GTEx, including authorization to release the patient’s medical records and social history, sequencing of the donor’s genome, and blanket consent for all future research using the donated tissue and resultant data. The donor and tissue information is available in Additional file 2: Table S1 in the Supplementary Appendix. For the independent mQTL validation (USC cohort), newborn blood spots from pediatric glioblastoma cases and controls (47 samples total) were obtained from the California Biobank, using information from the California Cancer and Vital Statistics registries. Genotype data for the 188 individuals were generated by GTEx, and for the other 47 samples, DNA extraction, preprocessing, and genotyping were performed as previously described [79] (see Additional file 1: Materials and Methods for more details).

### Target capture bisulfite sequencing and data processing

Out of 9926 CoRSIVs previously reported [37], we included only those within 3000 base pairs from the body of a gene present in the PubTator [80] compendium, using BEDTOOLS [81] software, yielding 4641 CoRSIVs as targets for capture. The goal of using PubTator was to focus not just on known genes but on those most likely to be associated with a measurable phenotypic outcome. Libraries were made using the Agilent SureSelect Methyl-seq library kit with modifications (Design ID: S3163502**)**. Capture design details and version history are available in Additional file 1: Materials and Methods. As for the data processing, Bisulfite-sequencing reads were trimmed using Trim Galore, then mapped to the human genome build UCSC hg38 using the Bismark aligner [82]. Uniquely mapped reads were retained for further analysis (see Additional file 1: Materials and Methods). Our CoRSIV-capture reagents are commercially available from Agilent Technologies, Inc.

### Evaluating genetic influences on CoRSIV methylation

Analysis of associations between CoRSIV DNA methylation and genetic variation in cis was performed relying on the Simes correction as described previously [44]. Using the EMatrixQTL R package [83], Spearman rank correlation was computed for all SNVs within 1Mb of each CoRSIV, and the Simes correction was applied. Simes-adjusted *p*-values for each CoRSIV were collected, and the false discovery rate (FDR) correction was applied across all CoRSIVs analyzed in each tissue, with significance achieved at FDR-adjusted *p*<0.05. To compare the summed total of mQTL detected at CoRSIVs vs. that reported by GoDMC [42], mQTL associations were identified with *P* < 10^−10^. This conservative *P* value was selected to avoid false positives, given the relatively small number of individuals in the GTEx CoRSIV analysis. To further evaluate genetic influence on CoRSIV methylation, we used a haplotype-based approach. Phased genotype data from GTEx were used to infer each individual’s haplotype within the haplotype block overlapping each CoRSIV and correlations between CoRSIV methylation and haplotype allele sum were assessed (see Additional file 1: Materials and Methods).

## Supplementary Information

**Additional file 1:** Supplementary Materials and Methods, Supplementary Figures.

**Additional file 2: Table S1.** GTEx Donor Information and Tissue Types. **Table S2.** CoRSIVs targeted for Bisulfite Capture Sequencing (hg38). **Table S3.** CoRSIV Capture Sequencing Data QC Metrics. **Table S4.** CoRSIVs which failed to meet coverage criteria in all libraries (hg38). **Table S5.** Inter-tissue Pearson correlation coefficients across six tissues (see Fig. 3 F). **Table S6.** CoRSIV average methylation data for those adequately covered in all six tissues (see Fig. 1 G). **Table S7.** Capture efficiency data do not assocociate with Fig. 1 G clustering. **Table S8.** Simes CoRSIV-SNV mQTL in GTEx Data (sorted by R-Squared). **Table S9.** Pearson correlation coefficients for haplotype allele sum vs. CoRSIV DNA methylation. **Table S10.** Simes CoRSIV-SNV mQTL in USC Data (sorted by R-Squared). **Table S11.** Pearson correlation coefficients for haplotype allele sum vs. CoRSIV DNA methylation USC Data. **Table S12.** Enrichment of repeat elements in Genic CoRSIVs vs. Controls. **Table S13.** Enrichment of repeat elements in Genic CoRSIV mQTL slope Neg. Vs. Pos. **Table S14.** Enrichment of repeat elements in R-squared Q4 CoRSIV mQTLs. vs. Q1 CoRSIV mQTLs (Genic). **Table S15.** Enrichment of repeat elements in Genic CoRSIVs Vs. HM450K. **Table S16.** Tajima's D Score comparison between CoRSIVs, Controls, tDMRs, HM450k. **Table S17.** CoRSIV mQTL SNV association with GWAS SNVs. **Table S18.** EPIC Array probes overlapping known SIV regions (hg38). (Note duplication across studies.).

## Data Availability

The raw target capture bisulfite sequencing data for the 807 GTEx tissues (188 individuals) have been deposited to the AnVIL repository [84]. Controlled access is administered through dbGaP (accession phs001746.v2.p1) [85]. The samples used in the mQTL validation analysis (USC cohort) are biospecimens from the California Biobank Program. Any uploading of genomic data and/or sharing of these biospecimens or individual data derived from these biospecimens would violate the statutory scheme of the California Health and Safety Code Sections 124980(j), 124991(b), (g), (h), and 103850 (a) and (d), which protect the confidential nature of biospecimens and individual data derived from biospecimens. Full results of our mQTL and haplotype-based analyses on the USC cohort are available in Additional file 2: Tables S10 and S11, respectively.
