# Supplementary material for: Systemic interindividual epigenetic variation in humans is associated with transposable elements and under strong genetic control
Source: Genome Biol. 2023 Jan 12;24:2. doi: 10.1186/s13059-022-02827-3 (PMC9835319; doi:10.1186/s13059-022-02827-3)
Supplement: Supplementary file 1 — Additional file 1: Supplementary Materials and Methods, Supplementary Figures. [file 13059_2022_2827_MOESM1_ESM.pdf]

Supplementary Materials for

Systemic interindividual epigenetic variation in humans is associated with transposable elements and under strong genetic control

Chathura J. Gunasekara, Harry MacKay, C. Anthony Scott, Shaobo Li, Eleonora Laritsky, Maria S. Baker, Sandra L. Grimm, Goo Jun, Yumei Li, Rui Chen, Joseph L. Wiemels, Cristian Coarfa, Robert A. Waterland

Correspondence to: waterland@bcm.edu, coarfa@bcm.edu

This PDF file includes:

Materials and Methods
Figs. S1 to S23

Other Supplementary Materials for this manuscript include the following:

Table	Content
Table S1	GTEx Donor Information and Tissue Types
Table S2	CoRSIVs targeted for Bisulfite Capture Sequencing (hg38)
Table S3	CoRSIV Capture Sequencing Data QC Metrics
Table S4	CoRSIVs which failed to meet coverage criteria in all libraries (hg38)
Table S5	Inter-tissue Pearson correlation coefficients across six tissues (see Fig. 3F)
Table S6	CoRSIV average methylation data for those adequately covered in all six tissues (see Fig. 1G)
Table S7	Capture efficiency data do not assocociate with Fig. 1G clustering
Table S8	Simes CoRSIV-SNV mQTL in GTEx Data (sorted by R-Squared)
Table S9	Pearson correlation coefficients for haplotype allele sum vs. CoRSIV DNA methylation
Table S10	Simes CoRSIV-SNV mQTL in USC Data (sorted by R-Squared)
Table S11	Pearson correlation coefficients for haplotype allele sum vs. CoRSIV DNA methylation USC Data
Table S12	Enrichment of repeat elements in Genic CoRSIVs vs. Controls
Table S13	Enrichment of repeat elements in Genic CoRSIV mQTL slope Neg. Vs. Pos.
Table S14	Enrichment of repeat elements in R-squared Q4 CoRSIV mQTLs. vs. Q1 CoRSIV mQTLs (Genic)
Table S15	Enrichment of repeat elements in Genic CoRSIVs Vs. HM450K
Table S16	Tajima's D Score comparison between CoRSIVs, Controls, tDMRs, HM450k
Table S17	CoRSIV mQTL SNV association with GWAS SNVs
Table S18	EPIC Array probes overlapping known SIV regions (hg38). (Note duplication across studies.)

Materials and Methods

CoRSIV Capture Design Versions

V.	Agilent Design ID	Design Date	Included Regions	Agilent Design Selections	Size (Mbp)
1.0	S3163502	23-Aug-2018	4641 CoRSIVs<br>Sex specific Chr Y regions	1x Tiling Density<br>Least Stringent Masking<br>Balanced Boosting	9.045
2.0	S3223244	16-Jul-2019	9926 CoRSIVs<br>SIV (*1, 2*)<br>ESS (*2*)<br>Sex-specific Chr Y regions<br>imprinting control regions (*3*)	1x Tiling Density<br>Least Stringent Masking<br>Balanced Boosting	19.843
3.0	S3295946	06-Aug-2020	9926 CoRSIVs<br>SIV(*1, 2*)<br>ESS (*2*)<br>Cell Composition estimation probes (*4, 5*)<br>Sex-specific Chr Y regions,<br>imprinting control regions(*3*)	1x Tiling Density<br>Least Stringent Masking<br>Optimized Performance<br>XT/XT2 boosting	21.958
3.1	S3332366	23-Feb-2021	9926 CoRSIVs<br>SIV(*1, 2*)<br>ESS (*2*)<br>Cell Composition estimation probes (*4, 5*)<br>Sex-specific Chr Y regions,<br>imprinting control regions(*3*)	1x Tiling Density<br>Least Stringent Masking<br>Optimized Performance<br>XT/XT2 boosting<br>Overnight Hybridization<br>Deleted ineffective baits from v.3.0.	21.130

Design of CoRSIV-capture reagent

Of the 9,926 CoRSIVs previously reported (*6*), to ensure adequate targeting we filtered to include only those within 3,000 base pairs (bp) from the body of a gene present in the Pubtator (*7*) compendium, using BEDTOOLS (*8*) software, yielding 4,641 CoRSIVs as targets for capture (Supplementary Table). For quality control purposes we included 10 regions on the Y chromosome to confirm the accurate biological sex of each sample. At each of the 4,641 CoRSIVs, the target region included flanking regions of 1,000 bp in each direction. We used the Agilent SureSelect online system to design a custom capture reagent, using the following options: balanced boosting, 1x tiling, and least stringent masking. Overall, our CoRSIV capture reagent (**Agilent Design ID: S3163502**) targeted 9.045 MB of the human genome (Supplementary Table 2), using 85,538 probes.

Library preparation, capture, and sequencing

Individual libraries were made using the Agilent SureSelect Methyl-seq library kit, with modification. In brief, 1ug of genomic DNA was subject to shearing to 150-200bp in size using a Covaris sonicator. After purification through AMpure XP beads, end repair and A-Tailing was carried out. Then, 5ul of 15uM methylated library adaptor (IDT) was ligated to each sample, and the product with a size of 250-450bp was selected through Ampure XP beads.

Twelve libraries were pooled in equal proportions for target enrichment following an Agilent protocol (Sureselect Methyl-seq target enrichment system for Illumina multiplexed sequencing). After hybridization with probes (Agilent SureSelect, custom design), Dynabeads MyOne streptavidin T1 beads were used to bind the library. After several round of washes, the bound DNA was eluted in 0.1N NaOH and subjected to Bisulfite treatment using the EZ DNA Methylation Gold kit (Zymo Research). Final library was generated by amplification using Sureselect Methyl-seq PCR Master Mix and P5, P7 primers (Illumina). Sequencing was performed using an Illumina Novaseq 6000 at the Functional Genomics core, Department of Molecular and Human Genetics, Baylor College of Medicine.

Data processing

Bisulfite-sequencing reads were trimmed using Trim Galore, then mapped to the human genome build UCSC hg38 using the Bismark aligner (*9*). Uniquely mapped reads were retained for further analysis. Duplicate reads were not removed, as recommended for capture experiments by the Bismark manual. CpG-level methylation was quantified using the Bismark pipeline. For each sample, average proportional DNA methylation was computed at each CoRSIV for which at least half of the CpGs were covered by at least 5x reads.

Quality control assessment

To determine the proportion of 'on-target' reads, only those that mapped completely within a target region were counted; capture efficiency was calculated as the fraction of on-target reads divided by all uniquely mapped reads. To confirm the accuracy of the biological sex of each sample, coverage of chromosome Y control regions was measured. Signal density plots were generated using the BEDTOOLS(*8*) software, with data reported as reads per million reads mapped (RPM), and visualized using Integrative Genome Viewer (IGV) software (*10*).

Assessment of inter-tissue correlations

At each CoRSIV, inter-tissue correlations of average proportional DNA methylation were computed for all tissue-pairs in which coverage requirements were satisfied in at least 10 individuals in both tissues. Pearson correlation was computed using the Python Scientific Library, with significance achieved at $p<0.05$. Inter-tissue correlation plots were visualized using the Python seaborn visualization library.

CoRSIV/tissue DNA methylation clustering analysis

To assess the similarity of DNA methylation profiles across donors and tissues, donors with CoRSIV capture data in at least 4 tissues were considered. Next, CoRSIVs with sufficient coverage across all donors and tissues were selected. Finally, CoRSIV-average proportional DNA methylation values for each sample were clustered using the Euclidean distance metric and the average linkage method, and visualized using the seaborn Python visualization library.

Cross-tissue analysis of gene expression vs. CoRSIV methylation

For each GTEx donor included in our analysis, tissue-specific gene expression profiles were downloaded from the GTEx data portal, expressed in transcripts per kilobase million (TPM). For each tissue, the analysis focused on CoRSIV associated genes expressed in that tissue (average TPM expression > 0.5). (Tibial nerve was not included in this analysis due to the small number of samples with RNA-seq data available.) Thyroid, lung, and cerebellum were considered 'target tissues'. For each CoRSIV-associated gene expressed in each target tissue, we calculated the Pearson correlation between CoRSIV average methylation in that tissue and gene expression in that tissue. We then asked if the same correlation was found between CoRSIV average methylation in a 'surrogate tissue' (blood or skin) and gene expression in the target tissue. Within each 'expression tissue' and 'methylation tissue' pair, p values were corrected for multiple hypothesis testing using the Benjamini Hochberg method, with significance achieved for adjusted p-value<0.05. Agreement of correlation between gene expression and DNA methylation between target tissue and surrogate tissues (statistically significant and in the same direction) was plotted in pie charts using GraphPad Prism. For specific CoRSIVs, scatterplots of tissue-specific gene expression vs. tissue-specific DNA methylation were generated using the seaborn Python visualization library.

mQTL Analysis using CoRSIV capture data on GTEx Samples

Analysis of associations between CoRSIV-average DNA methylation and genetic variation in cis was performed using a previously described strategy relying on the Simes correction (*11*). Rather than test for all significant mQTL associations, this approach conservatively tests whether, at each CoRSIV, there is evidence of mQTL. For each donor, single nucleotide variant (SNV) profiles computed by the GTEx consortium were downloaded in vcf format (dbGaP accession phs000424.v8.p2). SNVs reported in dbSNP and with a minor allele frequency (MAF) of at least 5% were selected for further analysis. mQTL analysis was conducted independently for each tissue. For each CoRSIV, the number of donors with both sufficient coverage in the capture experiment for a specific tissue and with a WGS SNV profile available was determined; for each tissue, CoRSIVs with data for at least 20 donors were selected for mQTL analysis. To harmonize our mQTL analysis with those based on the Illumina BeadArray data, CoRSIV-average proportional DNA methylation values were converted to M-values (*12*) prior to analysis. Spearman rank correlation was computed for all SNVs within 1mb of each CoRSIV, using the EMatrixQTL R package (*13*), and the Simes correction was applied. Simes adjusted p-values for each CoRSIV were collected, and the false discovery rate (FDR) correction was applied across all CoRSIVs analyzed in each tissue, with significance achieved at FDR-adjusted p-value<0.05. The R^2 variance explained by the linear model for each CoRSIV (in each tissue) was computed using the Python scientific library. For each significant mQTL association, a parametric analysis was carried out using using EMatrixQTL to determine the beta coefficient of the linear association between CoRSIV-average DNA methylation and the *cis* genetic variant.

Manhattan plots of mQTL associations were generated for each tissue and each CoRSIV using the R statistical system displaying all the mQTL candidates at p<0.001. Three-dimensional Manhattan plots of the significant mQTL associations across all CoRSIVS, capturing the distance between strongest associated SNV and CoRSIVs and the linear beta coefficient, were

generated using the plotly R library. A distribution of the beta linear coefficients across all significant mQTL associations in each respective tissue was generated using the R library.

Haplotype-based analysis using capture data on GTEx samples

SNVs reported in dbSNP and with a MAF of at least 5% were include in the haplotype-based analysis. PLINK 1.9 (*14, 15*) was used to identify haplotype blocks, with default parameters. Index SNVs were obtained by parsing the PLINK output for each individual block. Only CoRSIVs overlapping with haplotype blocks were considered for haplotype-based analysis. Further, only GTEx donors with a WGS profile were included, and within each tissue we considered only CoRSIVs with sufficient capture data on at least 20 donors. At each CoRSIV, the minor allele sum was computed across all the index SNVs for each donor, using the convention 0 for homozygous major allele, 1 for heterozygous SNV, and 2 for homozygous minor allele. Again, CoRSIV-average methylation values were transformed to M values. The Pearson correlation coefficient was computed between CoRSIV average DNA methylation and the minor allele sum of its overlapping haplotype block. Correction for multiple hypothesis testing was performed using the Benjamini-Hochberg correction, with significance achieved at FDR-adjusted p-value<0.05. Plots of DNA methylation at individual CoRSIVs vs. minor allele sum within overlapping haplotype blocks were generated using the Python scientific library.

Analyzing consistency of CoRSIV mQTL across tissues

Recurrence of significant mQTL for each CoRSIV across the 6 tissues was assessed in two ways. First, at the most stringent level (the SNV level), an mQTL SNV-CoRSIV pair was considered recurrent if the same Simes-adjusted SNV was identified, and the beta coefficient had the same sign, within two or more tissues. Considering the high linkage disequilibrium among multiple SNVs within a haplotype block, we also evaluated recurrence at the haplotype block level. At this level, an mQTL association for a CoRSIV was considered consistent across multiple tissues if the Simes-adjusted SNVs identified in two or more tissues fell within the same haplotype block, and the beta coefficient had the same sign. mQTL recurrence was plotted as heat maps using the R statistical system.

USC pediatric cohort – genotyping and CoRSIV capture bisulfite sequencing

Pediatric glioblastoma cases and controls were selected from the California Biobank, using information from the California Cancer and Vital Statistics registries. Cases were self-reported non-Latino whites born between 1982 and 2009, and subsequently diagnosed with glioblastoma (ICDO-3 code 9440). Controls were born in the same year with same gender and ethnic group as cases from anywhere in the state. Neonatal dried blood spots (approx 1.3 cm diameter) for each child were used for DNA extraction. DNA extraction, preprocessing and genotyping were performed as previously described (*16*). In brief, DNA was extracted from 1/3 of a dried blood spot with Genfind v3.0 (Beckman) reagents on an Eppendorf robot, followed by in house quality control procedures including nanodrop for purity and pico-green measurement for DNA quantity. Four hundred ng DNA was genotyped using the Affymetrix Axiom Precision Medicine Diversity Array (PMDA) at Thermo Affymetrix (San Jose CA), and SNP calls were extracted using Affymetrix Powertools. CoRSIV-capture bisulfite sequencing was performed using CoRSIV Capture v2.0 (Design ID: S3223244).

USC Pediatric cohort CoRSIV capture data processing

For the USC pediatric whole blood cohort, Trim galore software was used for the quality control of the reads, which were aligned to hg38 genome using Bismark aligner (*9*). De-duplication was not carried according the Bismark guidelines for target capture sequencing. Bismark methylation extractor was used to do the methylation calling. CpG Methylation levels were averaged across CoRSIVs with at least 10x coverage.

Independent analysis of USC pediatric samples for confirmation of CoRSIV mQTL and effects of local haplotype

CoRSIV capture bisulfite sequencing data on whole blood (newborn blood spots) were generated for 48 individuals from the USC pediatric cohort. One individual was removed from the analysis as a genetic outlier, leaving 47 samples for this analysis. Phased genotype data were generated, and SNVs reported in dbSNP and with a minor allele frequency (MAF) of at least 5% were selected for further analysis. CoRSIV-average DNA methylation values were converted to M-values (*12*) prior to analysis. Spearman rank correlation was computed for all SNVs within 1mb of each CoRSIV, using the EMatrixQTL R package (*13*), and the Simes correction was applied. Simes adjusted p-values for each CoRSIV were collected, and the false discovery rate (FDR) correction was applied across all CoRSIVs analyzed in each tissue, with significance achieved at FDR-adjusted p-value<0.05.

For the haplotype-based analysis, SNVs reported in dbSNP and with a MAF of at least 5% were included. PLINK 1.9 (*14, 15*) was used to identify haplotype blocks, with default parameters. Index SNVs were obtained by parsing the PLINK output for each individual block. At each CoRSIV, the minor allele sum was computed across all the index SNVs for each donor, using the convention 0 for homozygous major allele, 1 for heterozygous SNV, and 2 for homozygous minor allele. The Pearson correlation coefficient was computed between CoRSIV DNA methylation and the minor allele sum of its overlapping haplotype block. Correction for multiple hypothesis testing was performed using the Benjamini-Hochberg method, with significance achieved at FDR-adjusted p-value<0.05.

Comparison of CoRSIV mQTL with HM450K mQTL results from GoDMC

GoDMC (Min et al. (*17*)) computed mQTL using 33,000 individuals with DNA methylation data generated in the HM450 platform. As described above, mQTL was calculated for the GTEx CoRSIV capture data using matrixEQTL software, regressing DNA methylation M-value against the genotype (0,1,2). To compare the summed total of mQTL detected at CoRSIVs vs. that reported by GoDMC, mQTL associations were identified with $P < 10^{-10}$. This conservative P value was selected to avoid false positives, given the relatively small number of individuals in the GTEx CoRSIV analysis. With this cut-off, approximately 150,000 cis mQTL effects were detected in each study. Since methylation data were not available from GoDMC, methylation ranges (delta) were inferred by multiplying the slope of the linear model by 2 (x-axis of the genotype call). For each individual mQTL association, the product (delta)x(R^2) yields the absolute quantity of interindividual methylation variation explained by the SNV. This metric was summed across all mQTL effects in each data set.

Analysis of regional enrichments of transposable elements

To compare genomic enrichment of transposable elements flanking CoRSIVs vs. non-CoRSIV regions, repeat definitions encoded in the RepeatMasker track were downloaded from the UCSC genome browser build hg38. Repeats were analyzed at the level of repeat class, repeat class and repeat family and, finally, repeat class, repeat family, and repeat names. Only repeat sets with at least 10,000 entries were included in the enrichment analyses. CpG islands, defined by the UCSC genome browser on the human genome build UCSC hg38, were also downloaded. Analysis extended to +/- 50,000 bp relative to each CoRSIV or comparison region. To compare the differential enrichment of one repeat subset R between two sets of genomic intervals A and B, we used BEDTOOLS (*8*) to determine repeat overlap between R and A or between R and B, within each of 50 genomic windows, cumulatively stepping by 1,000 bp increments from 0bp to 50,000bp. Within each window, an odds-ratio and p-value were computed using the Fisher's exact test. Multiple testing correction was performed within each genomic window to adjust for the multiple tests performed at each repeat type, with significance achieved at an FDR-adjusted P < 0.01. Odds ratios (enrichments or depletions) surviving multiple testing correction were plotted across all repeat subsets and genomic windows using GraphPad Prism.

Evolutionary selection analysis

Selection scores computed using Tajima's D score (*18*) across CEU specimens profiled by the 1000 genomes project were downloaded (*19*). Selection scores compiled within a 30kb radius around CoRSIVs, control regions, tDMR regions, or 450k probes were plotted using the R statistical analysis system.

Enrichment of GWAS trait SNVs

We employed permutation testing to determine the extent to which CoRSIV mQTL SNVs (Simes SNVs) are enriched for trait-associated SNVs from the NHGRI GWAS catalogue (downloaded October 2020). For each of 8 manually curated trait categories (*20*), we generated a null distribution by randomly selecting one SNV from the GTEx database (MAF >= 0.05) within 1 Mb up- or downstream from the center of each CoRSIV. We then determined whether this randomly chosen SNV overlapped an NHGRI trait-associated SNV from that category. This process was repeated 10,000 times to yield a null distribution for each trait category. The numbers of actual overlaps between CoRSIV mQTL SNVs and NHGRI trait-associated SNVs were compared to these null distributions using one-proportion Z tests. Bonferroni-adjusted p-values for these tests are reported. Enrichment was defined as the number of actual overlaps between CoRSIV mQTL SNVs and NHGRI trait-associated SNVs divided by the mean of the null distribution.

CoRSIV +/- 20kb SNVs heritability enrichment with/without controlling for 53 baseline features

To evaluate heritability for a variety of traits (i.e., cancer and metabolic diseases) in CoRSIV +/-20kb SNVs, we used stratified linkage disequilibrium score regression (s-LDSC) (*21*) . First, the targeted CoRSIV regions were lifted to hg19 using the UCSC "liftover" software, and "bedtools" software was used to add the +/- 20kb flanking. CoRSIV +/- '20kb' distance was used because the majority of significant mQTL occurred within these regions. European

population plink files used by LDSC software (v. 1.0.1) were downloaded from the 1000 Genomes project.

In step 1, "make_annot.py" python script available in LDSC software was used to generate SNV annotation for CoRSIV +/- 20kb and 53 'baseline' (*21*) bed files, creating (.annot) files typically consisting of CHR, BP, SNP, and CM columns, followed by one column per annotation, with the value of the annotation for each SNP (0/1 for binary categories). Two separate sets of annotation files were generated for CoRSIV +/- 20kb with and without 53 'baseline' features.

In step 2, two annotation file sets generated in step1 were used to estimate annotation-specific LD scores using 1000 Genomes phase3 plink files and 1000G HapMap3 SNPs excluding MHC region in chr6 (according to the LDSC user manual). LDSC.py script with the following parameters suggested by the manual was used. --bfile flag points to the plink format fileset; The --l2 flag tells ldsc to compute LD Scores. The --ld-wind-cm flag tells lsdc to use a 1 cM window to estimate LD Scores.

In step 3, to calculate partitioned heritability for different traits, summary GWAS statistics were downloaded for 4 cancer and 12 metabolic disease categories from (https://alkesgroup.broadinstitute.org/LDSCORE/all_sumstats/). The LDSC.py script with --h2 flag compute partitioned heritability and outputs enrichment scores, for CoRSIV +/- 20kb regions (with/without controlling for 53 baseline features) for each trait.

Supplementary Figures

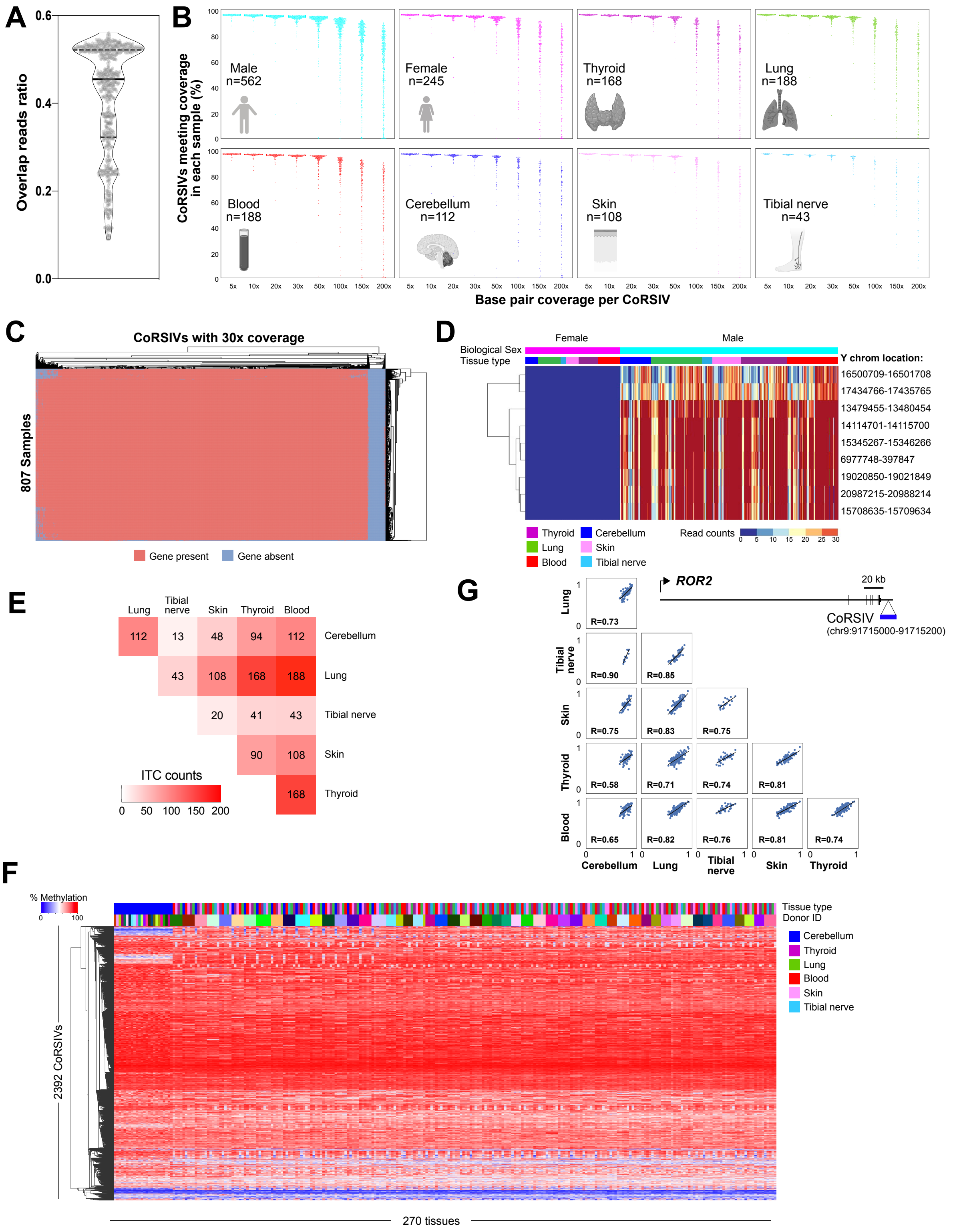

Fig. S1. CoRSIV capture efficiency, quality control, and inter-tissue correlation in DNA methylation. (A) Violin plot shows, for each of 807 samples, the proportion of reads that were on target (i.e. completely within a target region). **(B)** Percentage of CoRSIVs for which target-capture bisulfite sequencing achieved various read depths, by sex, and by tissue type. Each point represents a sample; numbers of samples are shown. **(C)** Dichotomous heat map showing which of 4,483 CoRSIVs (columns) are covered at ≥30x depth in each of 807 samples (rows). A small fraction of CoRSIVs proved difficult to capture across all samples. **(D)** Read-depth across a panel of Y-chromosome probes confirms correct biological sex for all 807 samples (quality control). **(E)** Heat map shows numbers of samples available to calculate inter-tissue correlations. **(F)** For the 270 tissue samples from 53 donors with data on at least 5 tissues (including cerebellum), unsupervised hierarchical clustering of methylation data at 2,340 fully informative CoRSIVs organizes mainly by donor, but also forms a minor cerebellum cluster (left hand side). **(G)** Inter-tissue correlation plots for a CoRSIV near *ROR2* show that, despite higher methylation in cerebellum relative to other tissues, high inter-tissue correlations are maintained.

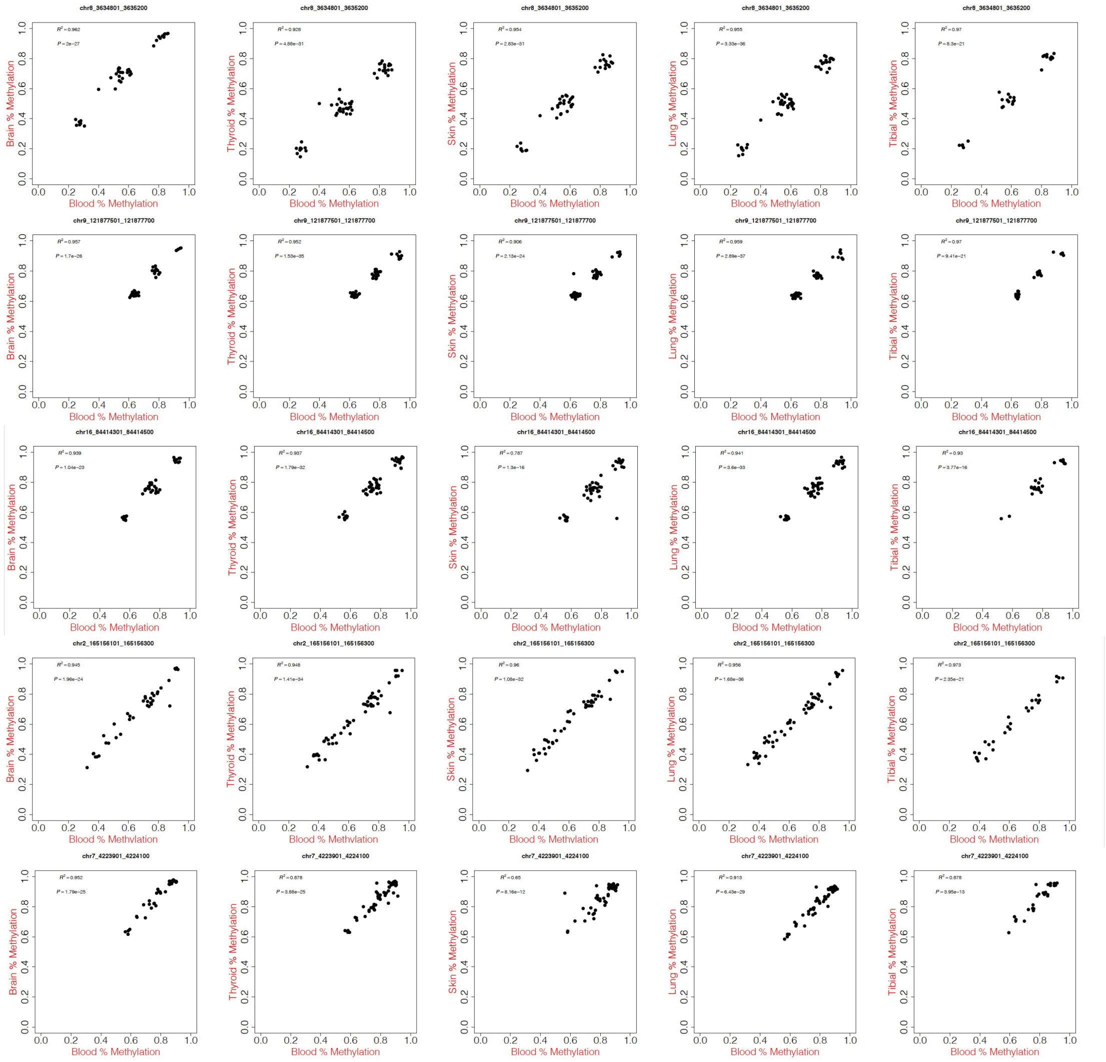

Fig. S2. Inter-tissue correlation (ITC) plots show that methylation in blood is associated with that in brain, thyroid, skin, lung, and tibial nerve. Each row shows data on one CoRSIV. The first three and last two rows show CoRSIVs with three modes of methylation, and a uniform distribution, respectively (the most common patterns observed). For ITC plots on all the CoRSIVs see (five tissues vs. blood).

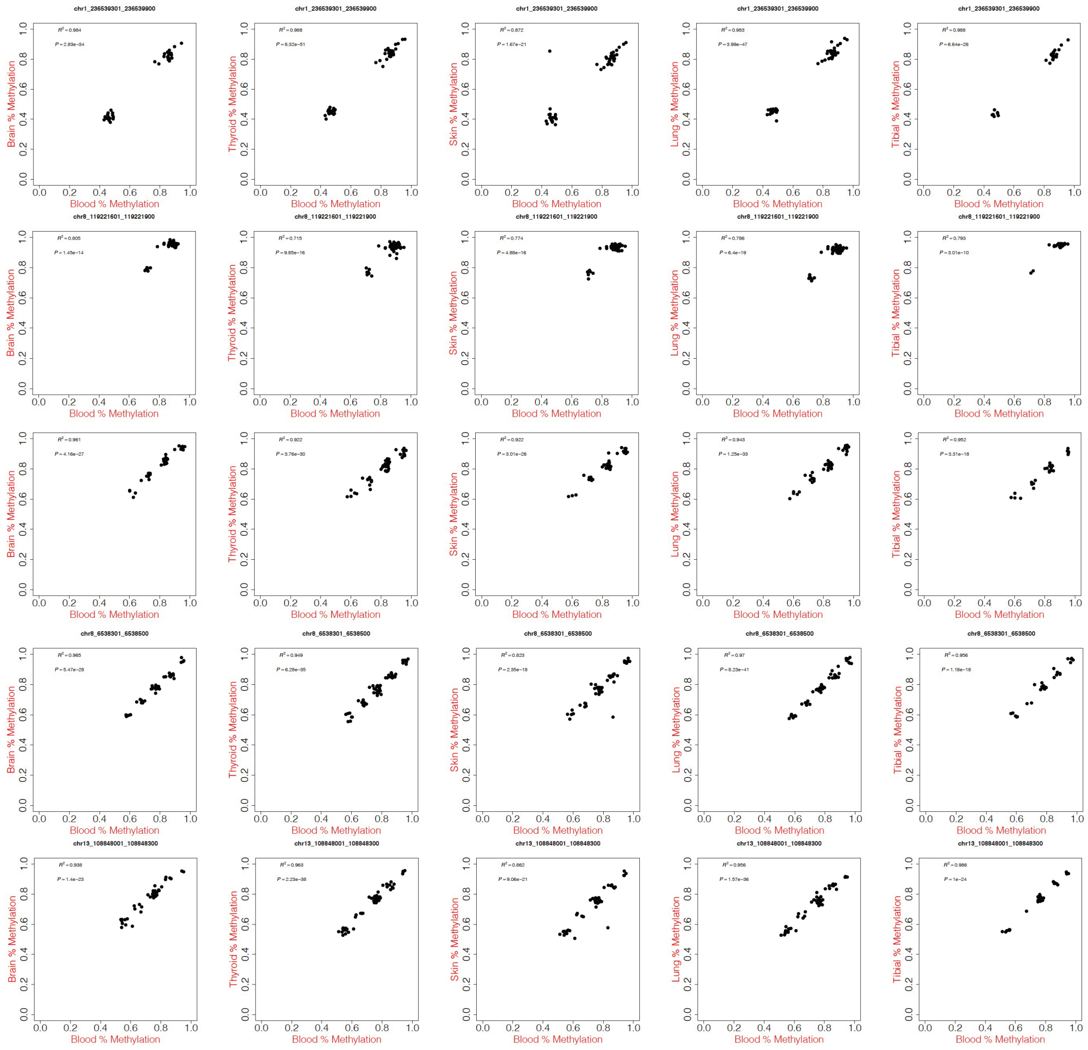

Fig. S3. Inter-tissue correlation (ITC) plots show that methylation in blood is associated with that in brain, thyroid, skin, lung, and tibial nerve. Each row shows data on one CoRSIV. These plots show examples of infrequently observed patterns including two, four, or five discrete modes. For ITC plots on all the CoRSIVs see (five tissues vs. blood).

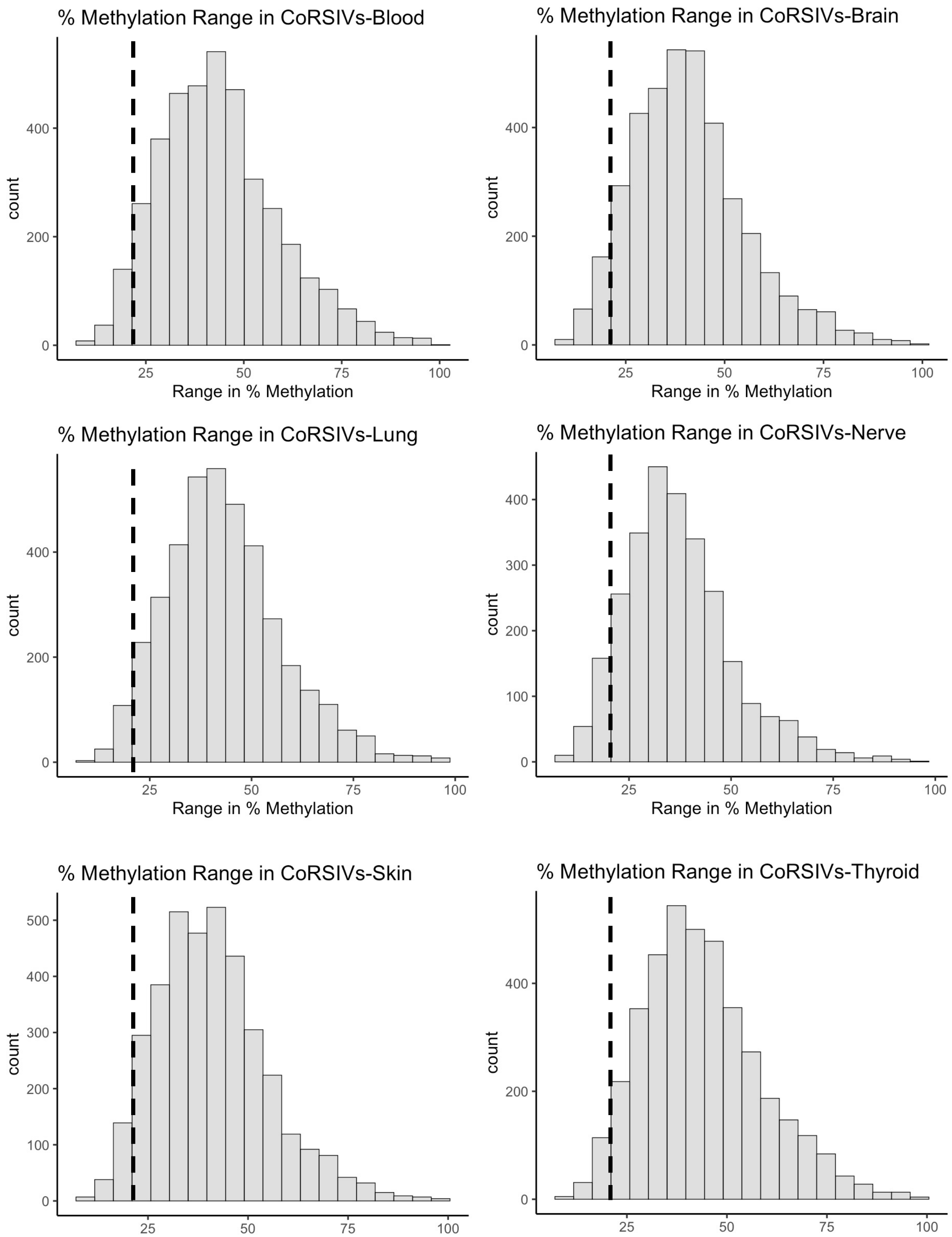

Fig. S4. CoRSIVs exhibit high interindividual variation in every tissue examined. Each plot shows the distribution of interindividual methylation range across 4,086 CoRSIVs. In all six tissues, nearly all CoRSIVs show an interindividual range > 20% (dashed line).

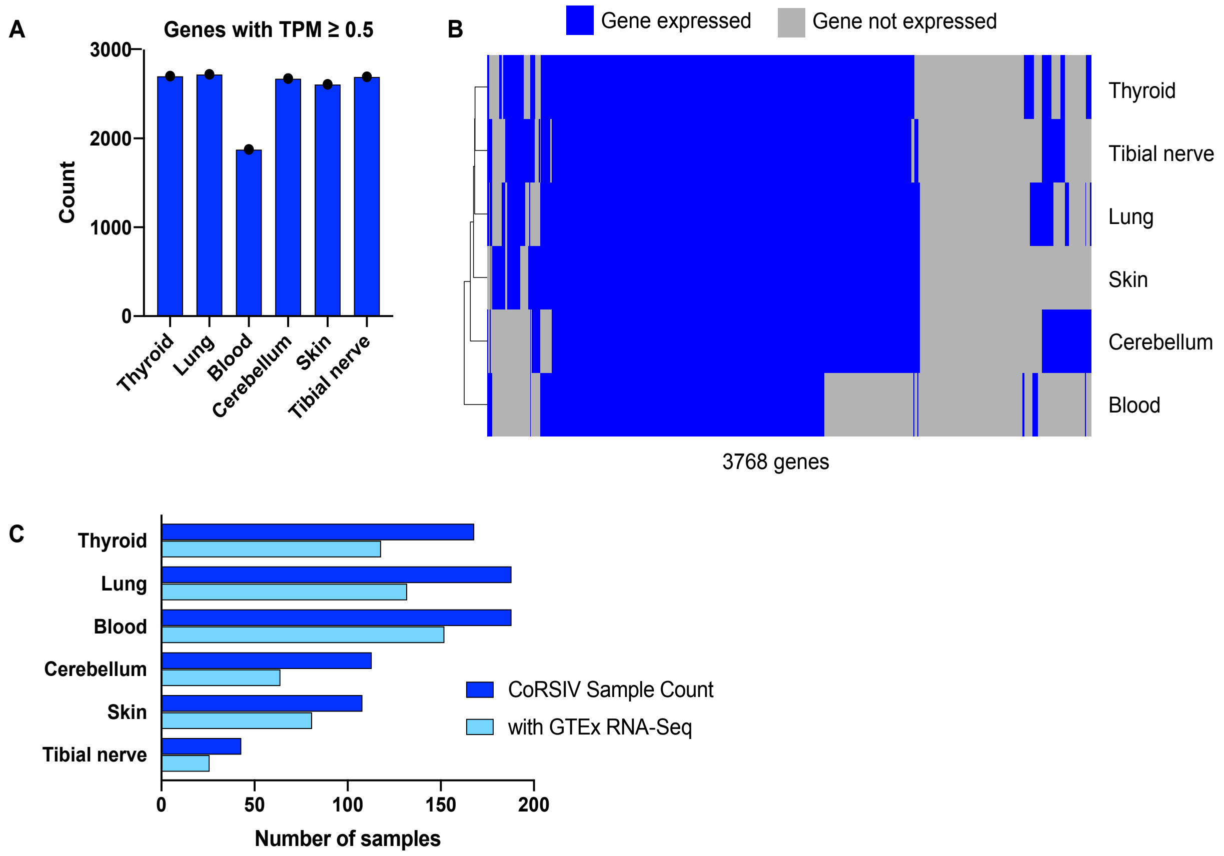

Fig. S5. Supporting data for cross-tissue analysis of methylation and gene expression. (A) Numbers of CoRSIV-associated genes expressed (transcripts per million (TPM) >= 0.5) in each tissue type. **(B)** Heat map of expressed genes across the six tissues. **(C)** Except for tibial nerve, both methylation and gene expression data were available for more than 60 individuals.

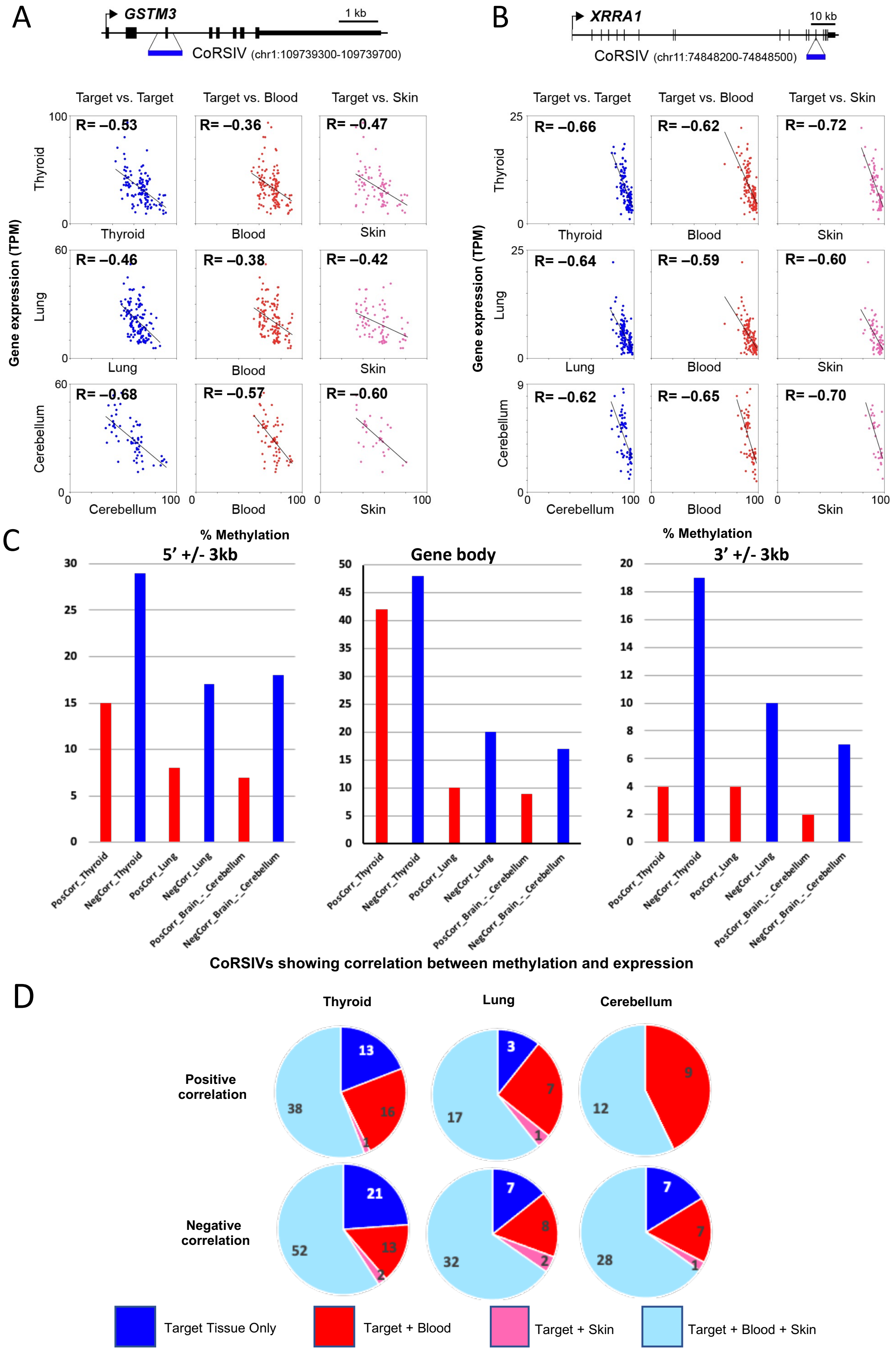

A
GSTM3
1 kb
CoRSIV (chr1:109739300-109739700)
Target vs. Target
Target vs. Blood
Target vs. Skin
R= –0.53
R= –0.36
R= –0.47
R= –0.46
R= –0.38
R= –0.42
R= –0.68
R= –0.57
R= –0.60
Gene expression (TPM)
Thyroid
Lung
Cerebellum
Blood
Skin
B
XRRA1
10 kb
CoRSIV (chr11:74848200-74848500)
R= –0.66
R= –0.62
R= –0.72
R= –0.64
R= –0.59
R= –0.60
R= –0.62
R= –0.65
R= –0.70
% Methylation
C
5' +/- 3kb
Gene body
3' +/- 3kb
PosCorr_Thyroid
NegCorr_Thyroid
PosCorr_Lung
NegCorr_Lung
PosCorr_Brain_-_Cerebellum
NegCorr_Brain_-_Cerebellum
CoRSIVs showing correlation between methylation and expression
D
Thyroid
Lung
Cerebellum
Positive correlation
Negative correlation
Target Tissue Only
Target + Blood
Target + Skin
Target + Blood + Skin

Fig. S6. CoRSIV methylation in blood or skin predicts expression of associated genes in less accessible tissues. (**A)** Within- and between-tissue correlations of *GSTM3* expression vs. DNA methylation at a CoRSIV within *GSTM3*. (**B)** Within- and between-tissue correlations of *XRRA1* expression vs. DNA methylation at a CoRSIV within *XRRA1*. **(C)** Number of CoRSIVs with positive and negative correlations in three tissues according to location of CoRSIV with respect to transcription start site (5'), gene body, and transcription end site (3'). (**D)** Thyroid , lung, and cerebellum are considered target tissues, and blood and skin are surrogate tissues. Across all CoRSIV-gene pairs with significant correlations between CoRSIV methylation and expression of an associated gene in a target tissue, over 75% show the same correlation when methylation in blood is used as surrogate; using skin as surrogate, over 60% do.

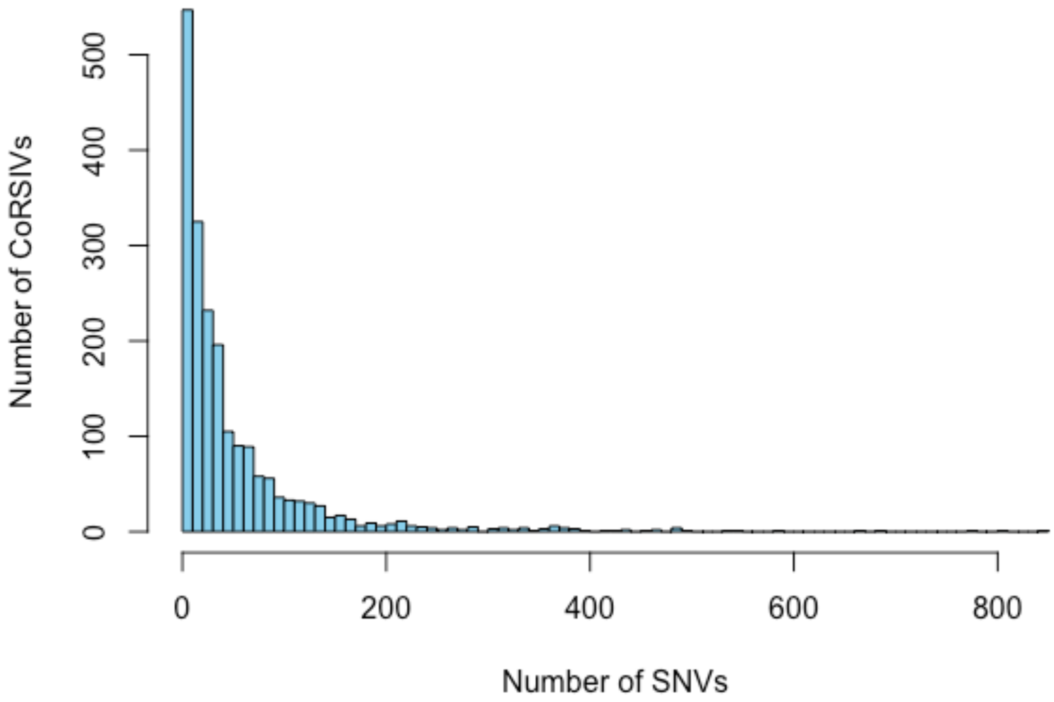

Fig. S7. Histogram of the number of genetic variants (SNVs) influencing methylation at each CoRSIV (mQTL P <10^{-10}).

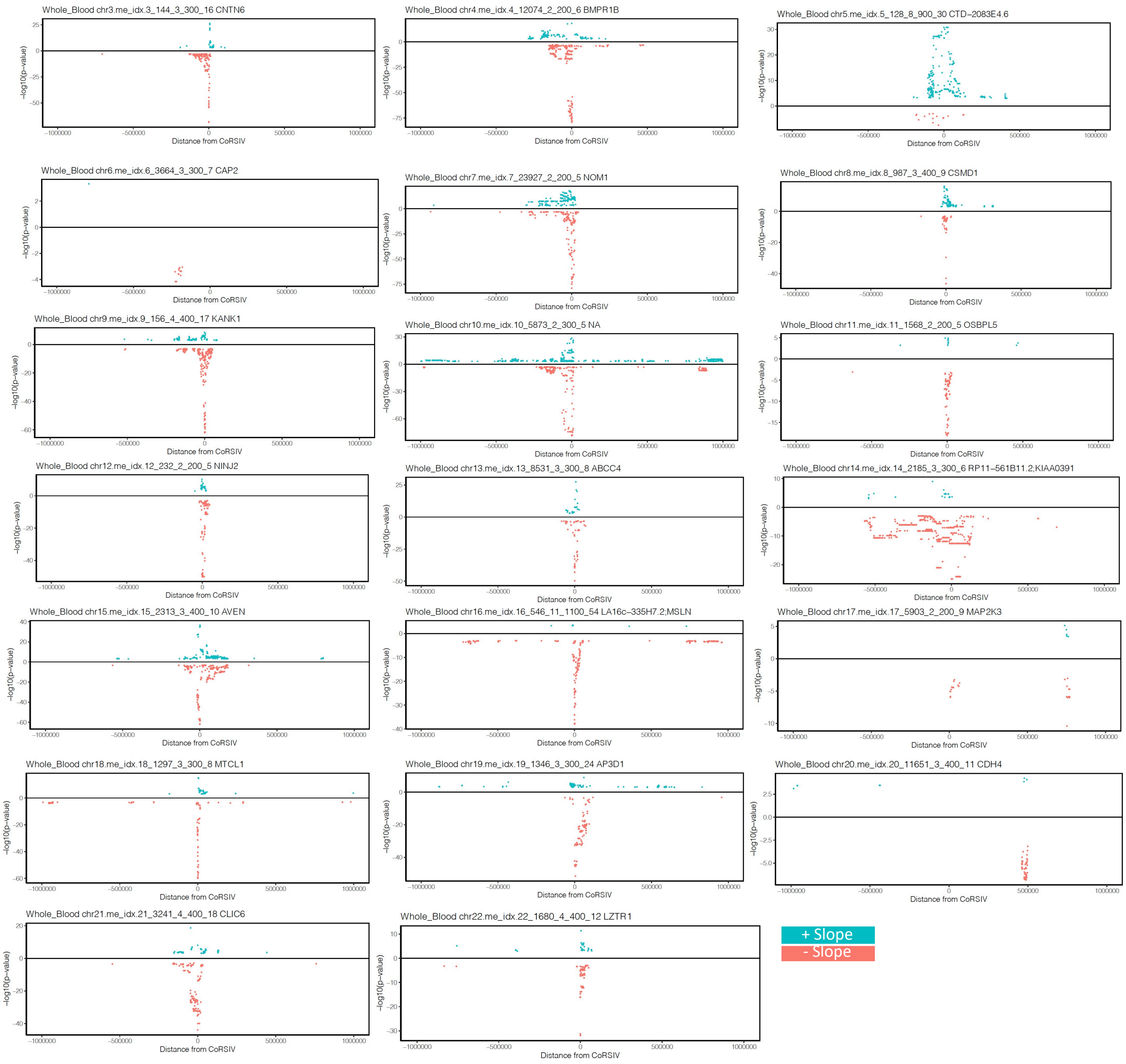

Fig. S8. Representative Manhattan plots of associations at individual CoRSIVs, in blood. Significant associations are shown for all SNVs within 1Mb of each CoRSIV; positive and negative beta coefficients are plotted in blue and red, respectively.

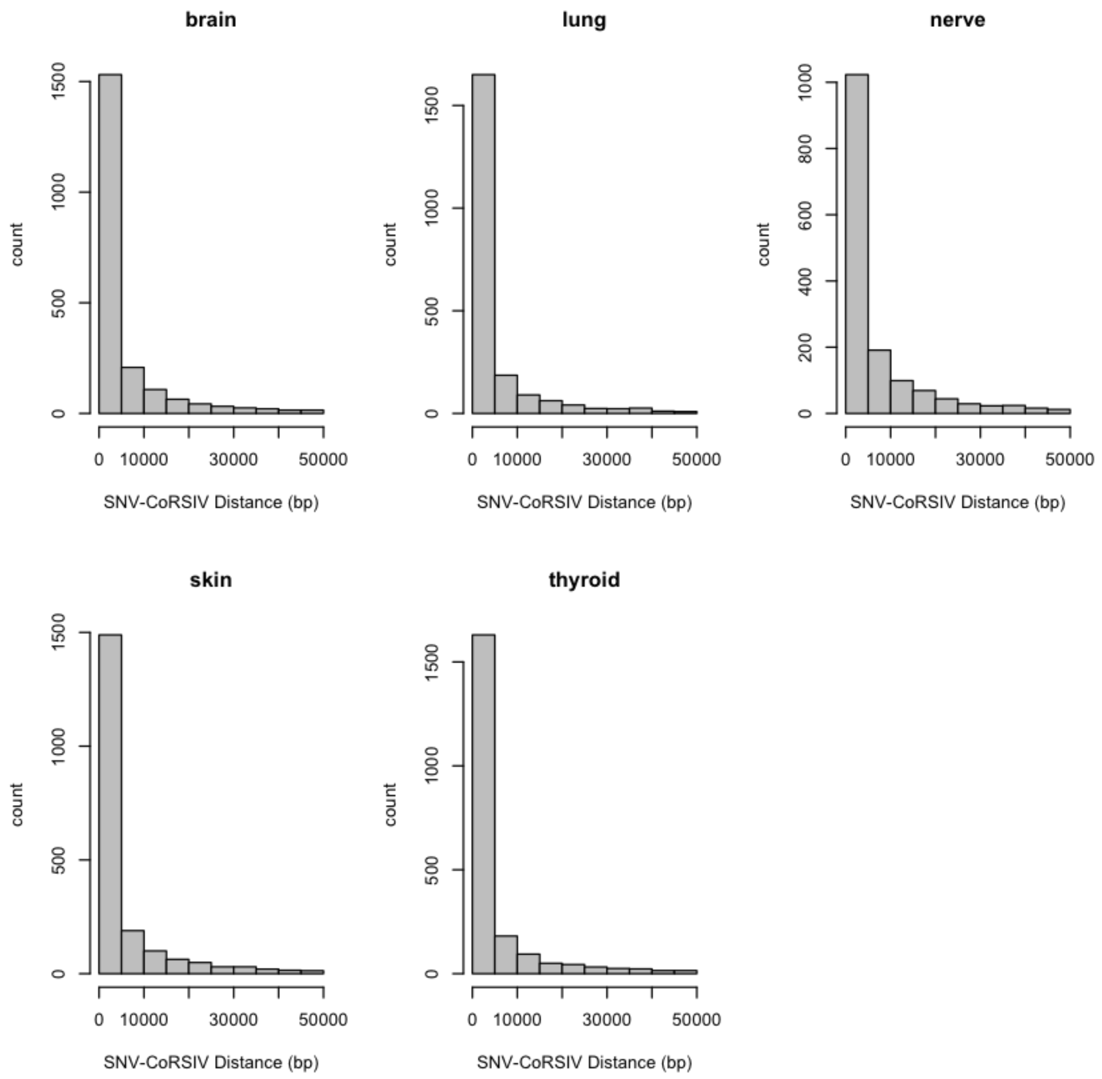

Fig. S9. Distribution of distances between CoRSIVs and corresponding Simes SNVs, in five different tissues. All appear nearly identical to the distribution in blood (Fig. 2C). The majority of Simes SNVs are within 10 kb of each CoRSIV.

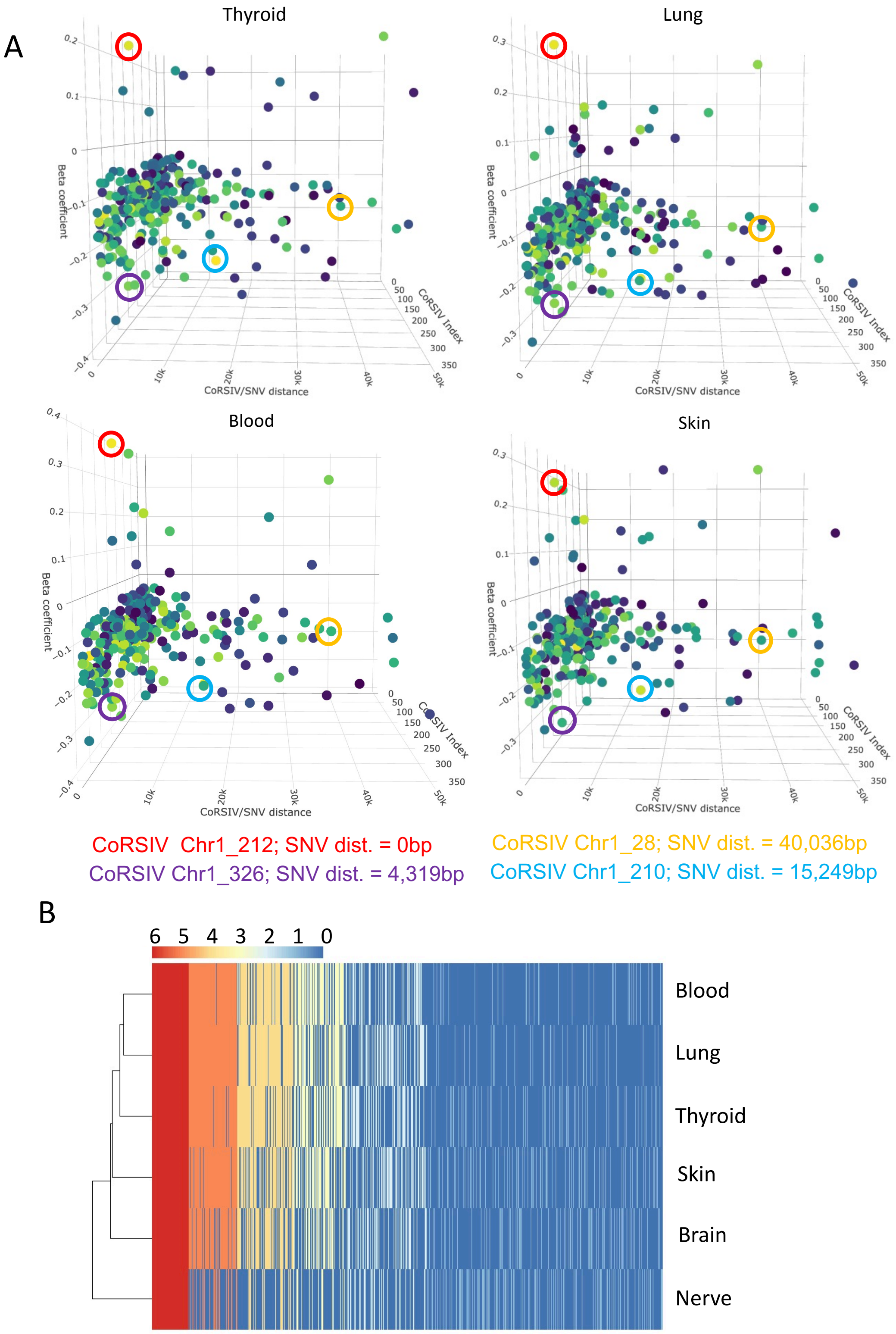

Fig. S10. Concordance of mQTL effects across different tissues. (A) Three-dimensional Manhattan plots for chromosome 1 show four examples (circled) of CoRSIVs at which the exact same SNV was independently identified as the Simes SNV in thyroid, lung, blood, and skin. **(B)** For each of 4,086 CoRSIVs, heat map depicts the number of tissues in which the same SNV is identified as the Simes SNV.

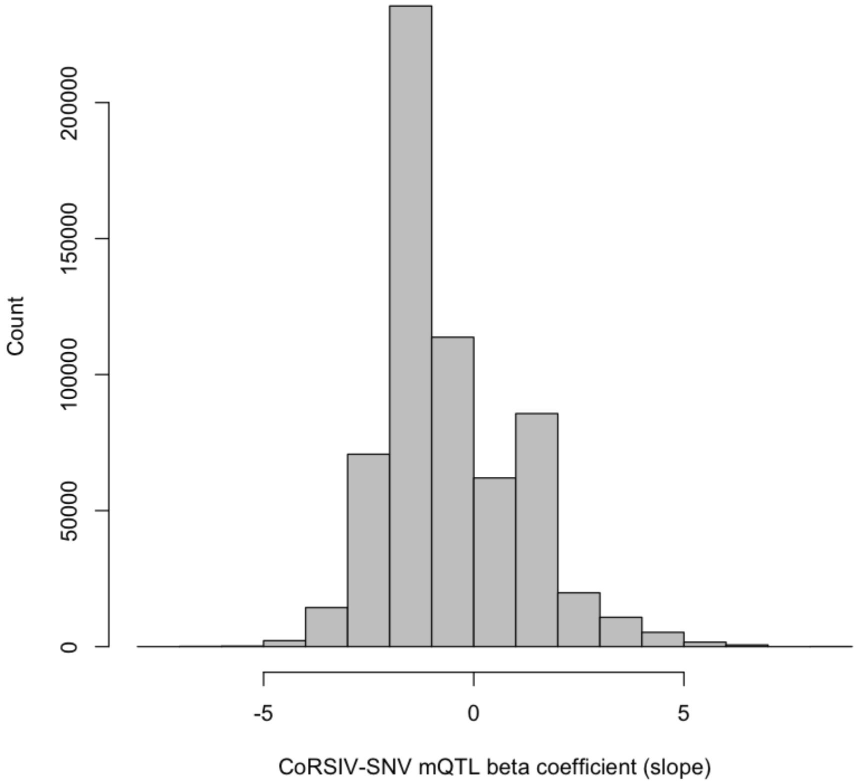

Fig. S11. All mQTL associations at CoRSIVs are biased toward negative beta coefficients. Shown is the distribution of all 146,698 mQTL associations detected within 1Mb of CoRSIVs ($P < 10^{-10}$) in blood. Similar to that of Simes SNVs (Fig. 2F), the bias toward negative beta coefficients is obvious.

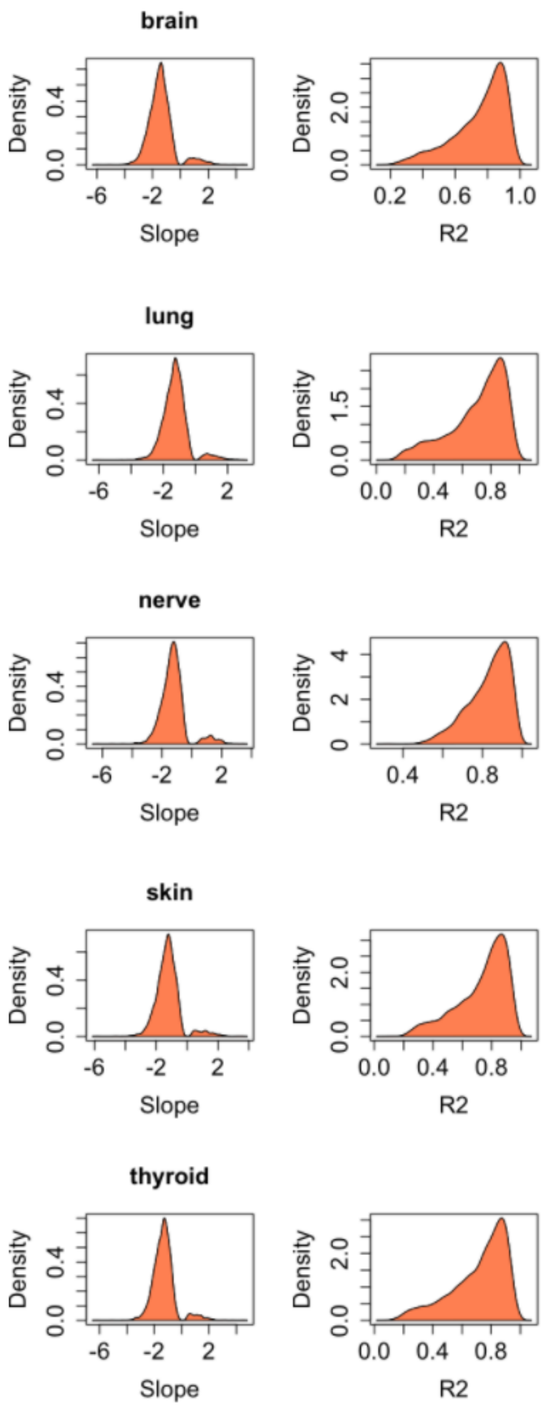

Fig. S12. Distribution of Simes mQTL slope (i.e. beta coefficient) and R^2 (goodness of fit) for brain, lung, tibial nerve, skin and thyroid are strikingly similar to those obtained for blood (Fig. 2 F, I).

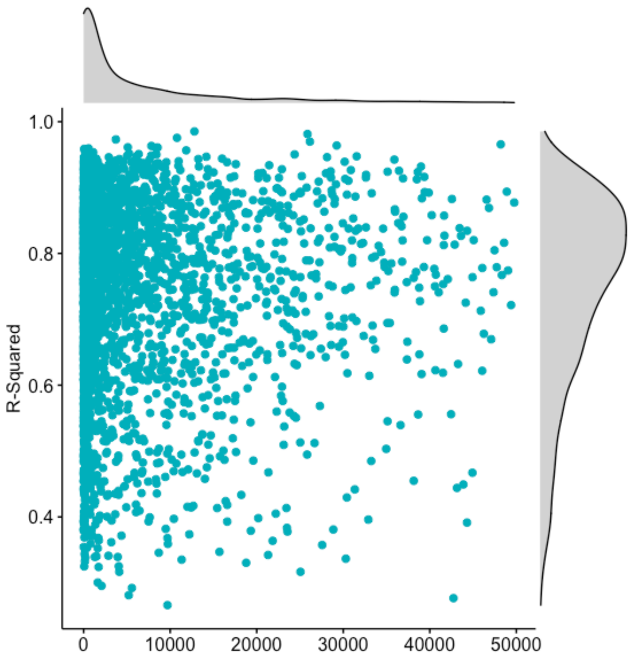

Fig. S13. Scatter plot of R-squared (goodness of fit) vs. CoRSIV-SNV distance for Simes mQTLs. The bias toward high R^2 mQTL effects is observed even at considerable CoRSIV-SNV distances.

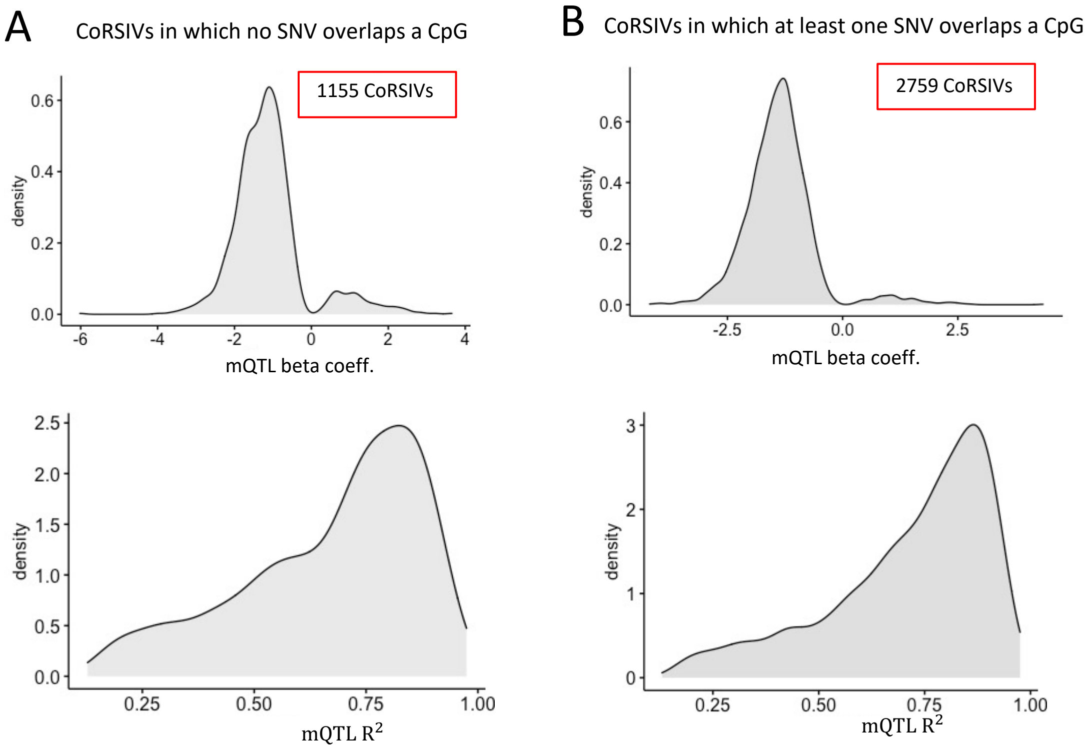

Fig. S14. Neither the bias toward negative beta coefficients nor the tendency for high R^2 of mQTL effects at CoRSIVs is explained by SNVs overlapping CpGs within CoRSIVs. (A) The set of 1155 CoRSIVs with no such overlaps. **(B)** The set of 2759 CoRSIVs for which at least on SNV overlaps a CpG within the CoRSIV. In both cases, the biases toward negative beta coefficients and high R^2 are observed.

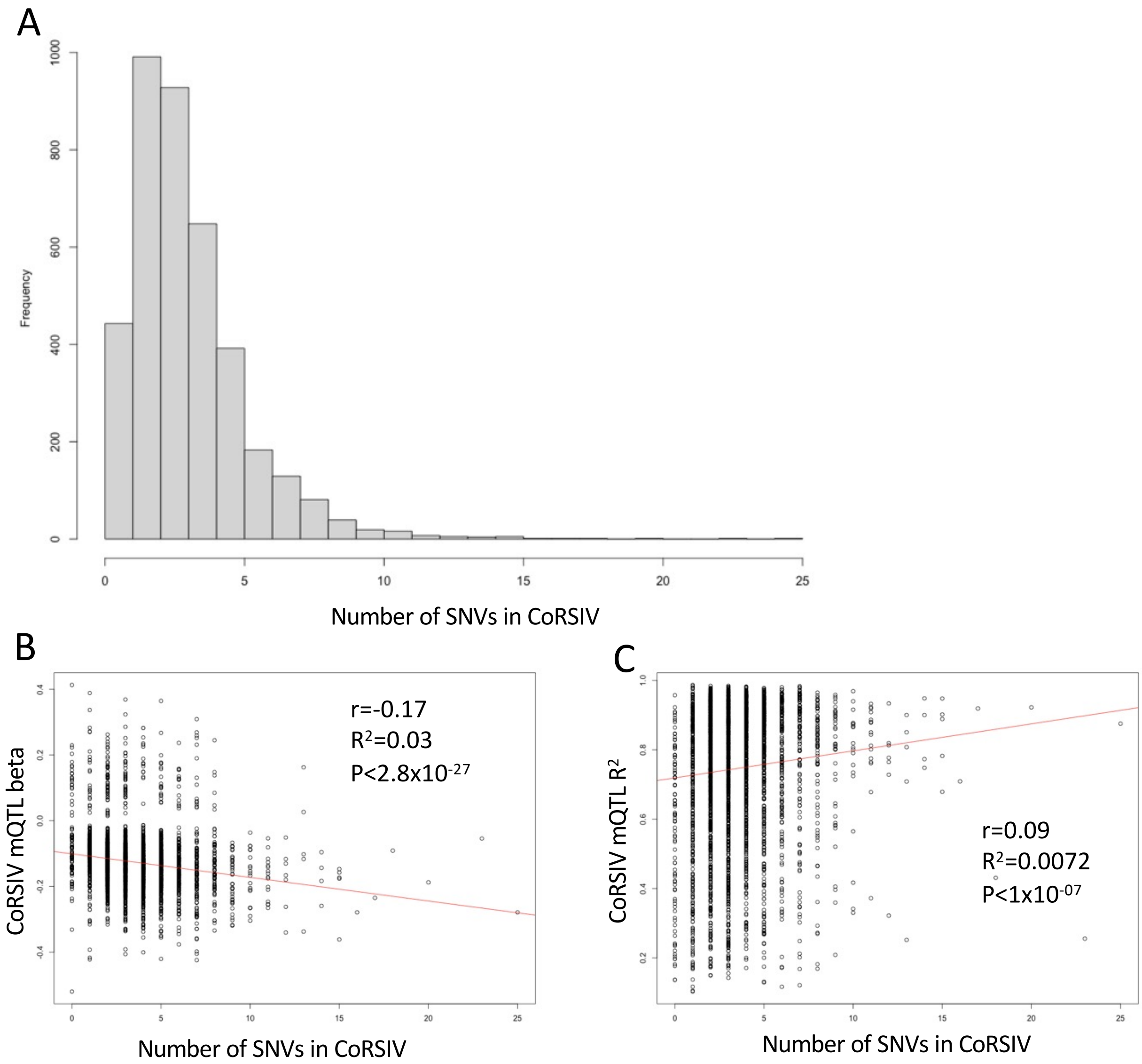

Fig. S15. SNVs within CoRSIVs do not explain either the bias toward negative beta coefficients or the strong R^2 of mQTL effects at CoRSIVs. (A) Distribution of the number of SNVs detected within each of 4,086 CoRSIVs. **(B)** CoRSIV mQTL beta coefficient is only weakly associated with the number of SNVs in each CoRSIV. **(C)** CoRSIV mQTL R^2 is only weakly associated with the number of SNVs in each CoRSIV.

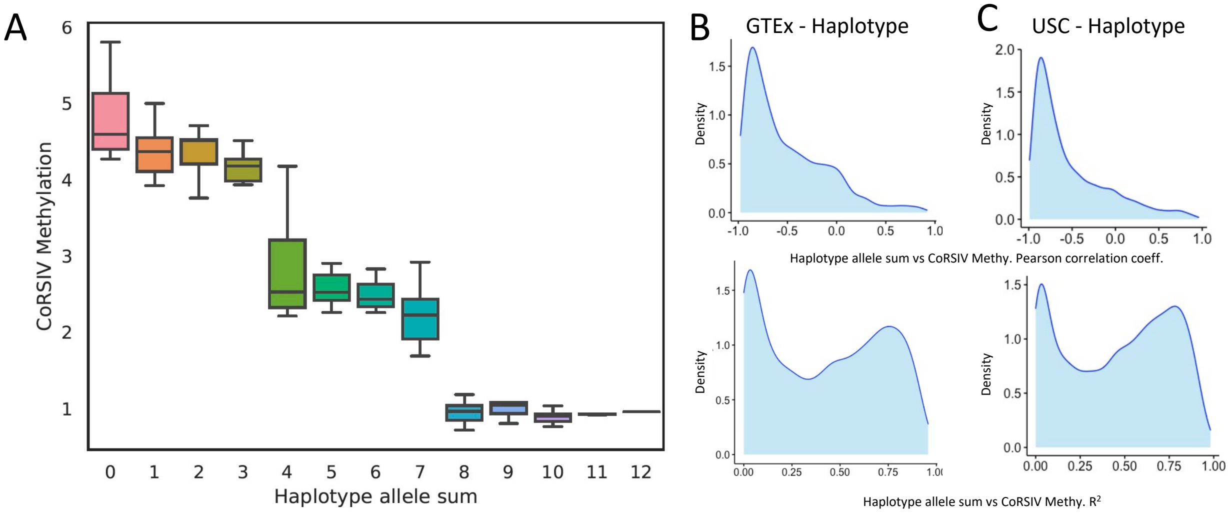

Fig. S16. Haplotype-based approach to assess local genetic influences on CoRSIV methylation. **(A)** Example of association of average methylation in blood at one CoRSIV (chr22:50677397-50683133) vs. haplotype allele sum (sum of minor alleles in each individual) for its overlapping haplotype block. Shown are data on 170 individuals grouped by haplotype allele sum. **(B)** Top: Distribution of Pearson correlation coefficients for all such associations (CoRSIV methylation vs. haplotype allele sum) across 4,471 CoRSIVs assessed in 188 GTEx donors. As in the mQTL analysis (Fig. 2F), negative coefficients predominate. Bottom: Distribution of R^2 (goodness of fit) across all such associations in GTEx donors. Local haplotype explains much of the variance in methylation. **(C)** Independent corroboration in USC cohort. Top: Distribution of Pearson correlation coefficients for all such associations (CoRSIV methylation vs. haplotype allele sum) across 4,471 CoRSIVs studied in 47 newborns in USC cohort. Bottom: Distribution of R^2 across all such associations in USC cohort.

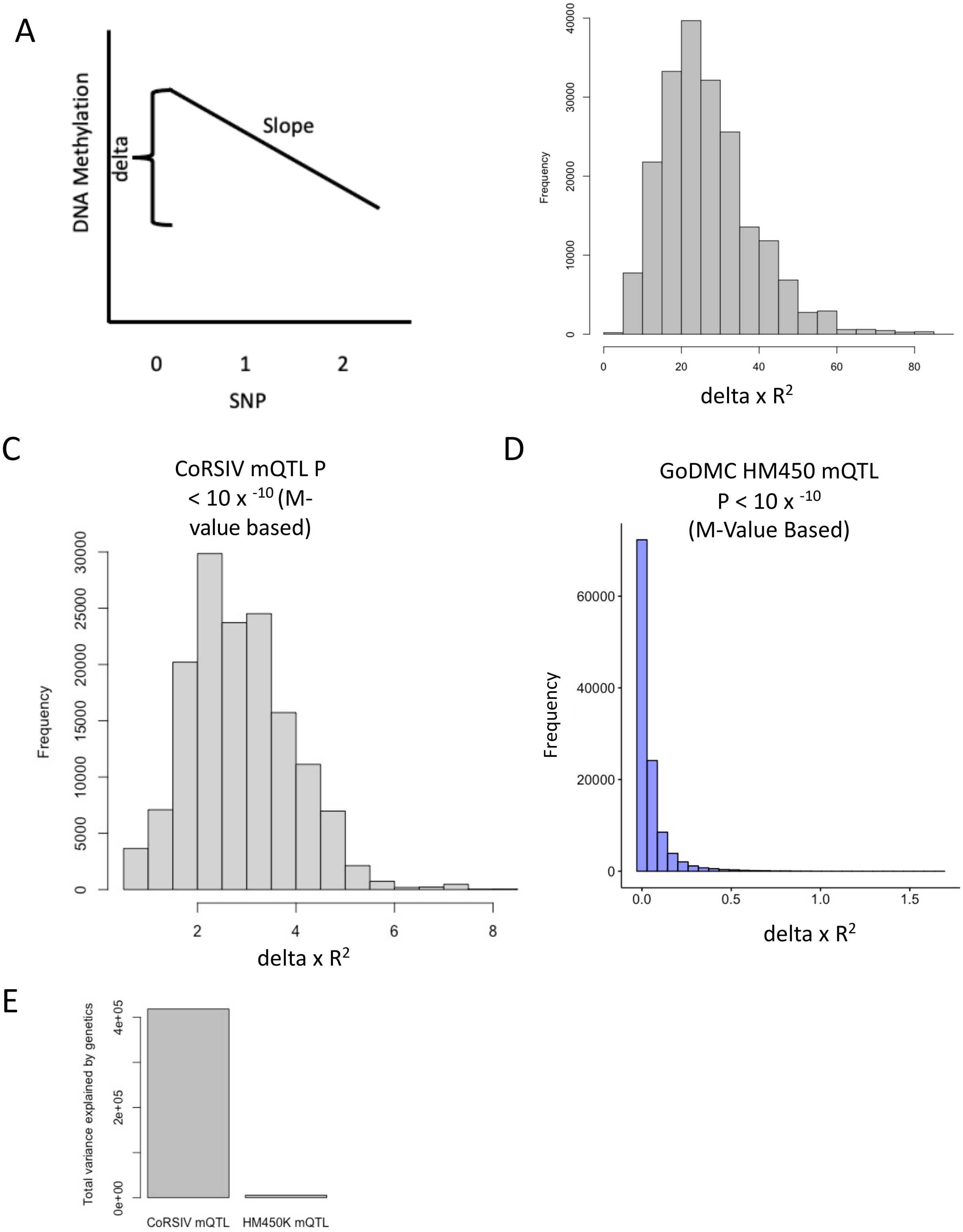
A
DNA Methylation
delta
Slope
0
1
2
SNP
Frequency
0
10000
20000
30000
40000
0
20
40
60
80
delta x R2
C
CoRSIV mQTL P
< 10 x -10 (M-
value based)
Frequency
0
5000
10000
15000
20000
25000
30000
2
4
6
8
delta x R2
D
GoDMC HM450 mQTL
P < 10 x -10
(M-Value Based)
Frequency
0
20000
40000
60000
0.0
0.5
1.0
1.5
delta x R2
E
Total variance explained by genetics
0e+00
2e+05
4e+05
CoRSIV mQTL
HM450K mQTL

Fig. S17. Approach for comparing total quantity of mQTL across different data sets. **(A)** With exactly 3 possible genotypes at every SNV, the methylation difference (delta) associated with each SNV can be easily determined from the beta coefficient (slope) of the mQTL association. **(B)** The product (delta)x(R^2) measures the total amount of variation in methylation that is determined by the SNV genotype. Distribution of (delta)x(R^2) for all 146,698 mQTL associations ($P < 10^{-10}$) across 2,738 CoRSIVs in blood of 188 individuals. **(C)** The same distribution, recalculated using M-values. **(D)** Distribution of (delta)x(R^2) for all 154,527 mQTL associations ($P<10^{-10}$) detected using the HM450 platform to study blood of 33,000 individuals (GoDMC data – Min et al 2021 Nat. Genetics). Although the calculations were performed the same way as in (C), note the very different x-axis scales. **(E)** Area under the curve in (C) vs. that in (D). The summed total variance in methylation explained by cis mQTL at CoRSIVs is 72 fold greater than that detected in the GoDMC report.

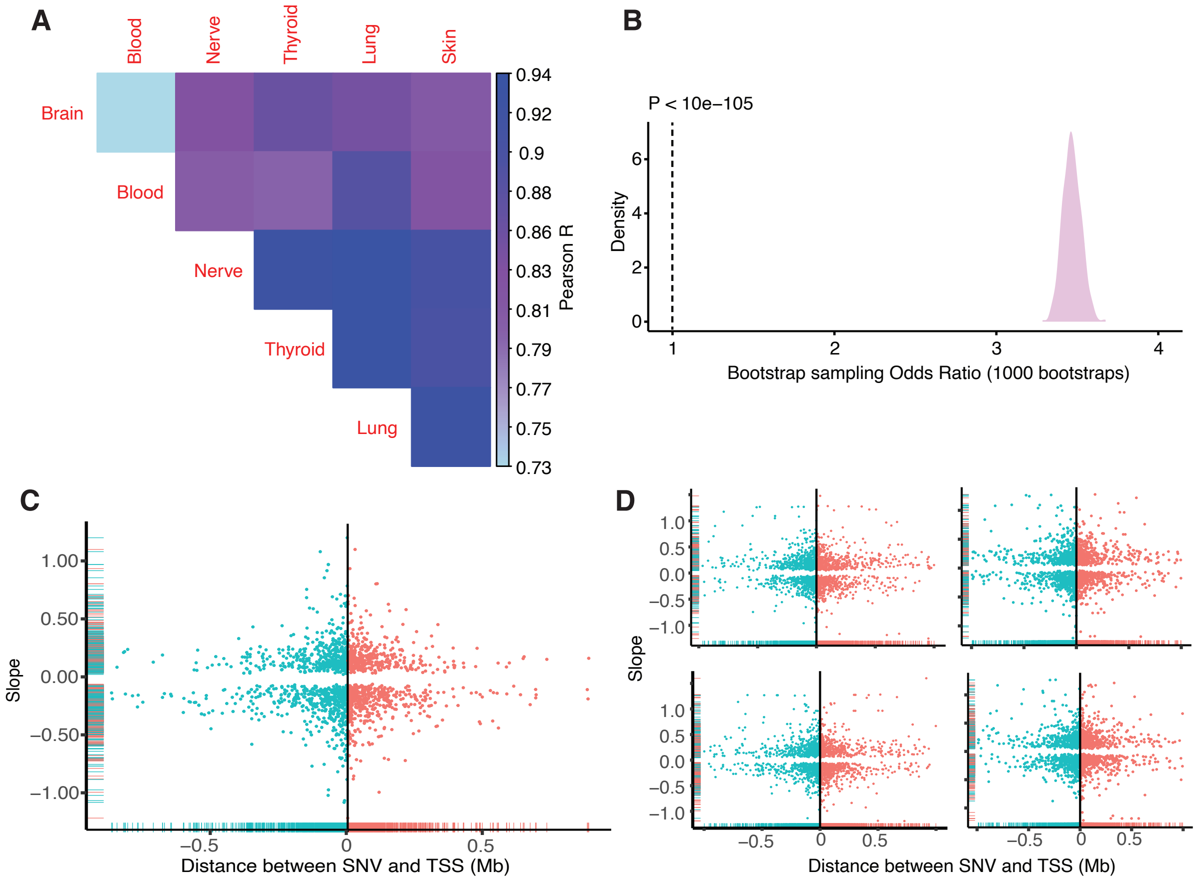

Fig. S18. Characteristics of eQTLs overlapping Simes SNVs. (A) Pearson correlation between eQTL slopes across 5 tissues. **(B)** Enrichment of Simes SNVs in eQTL compared to bootstrapped eQTLs from CoRSIV +/- 50kb flanking regions. Fisher test P-values < $1x10^{-105}$. **(C)** eQTL slope vs. distance between SNV and TSS for Simes SNVs. **(D)** eQTL slope vs. distance between SNV and TSS for bootstrapped eQTLs (four representative plots from 1000 bootstraps are shown).

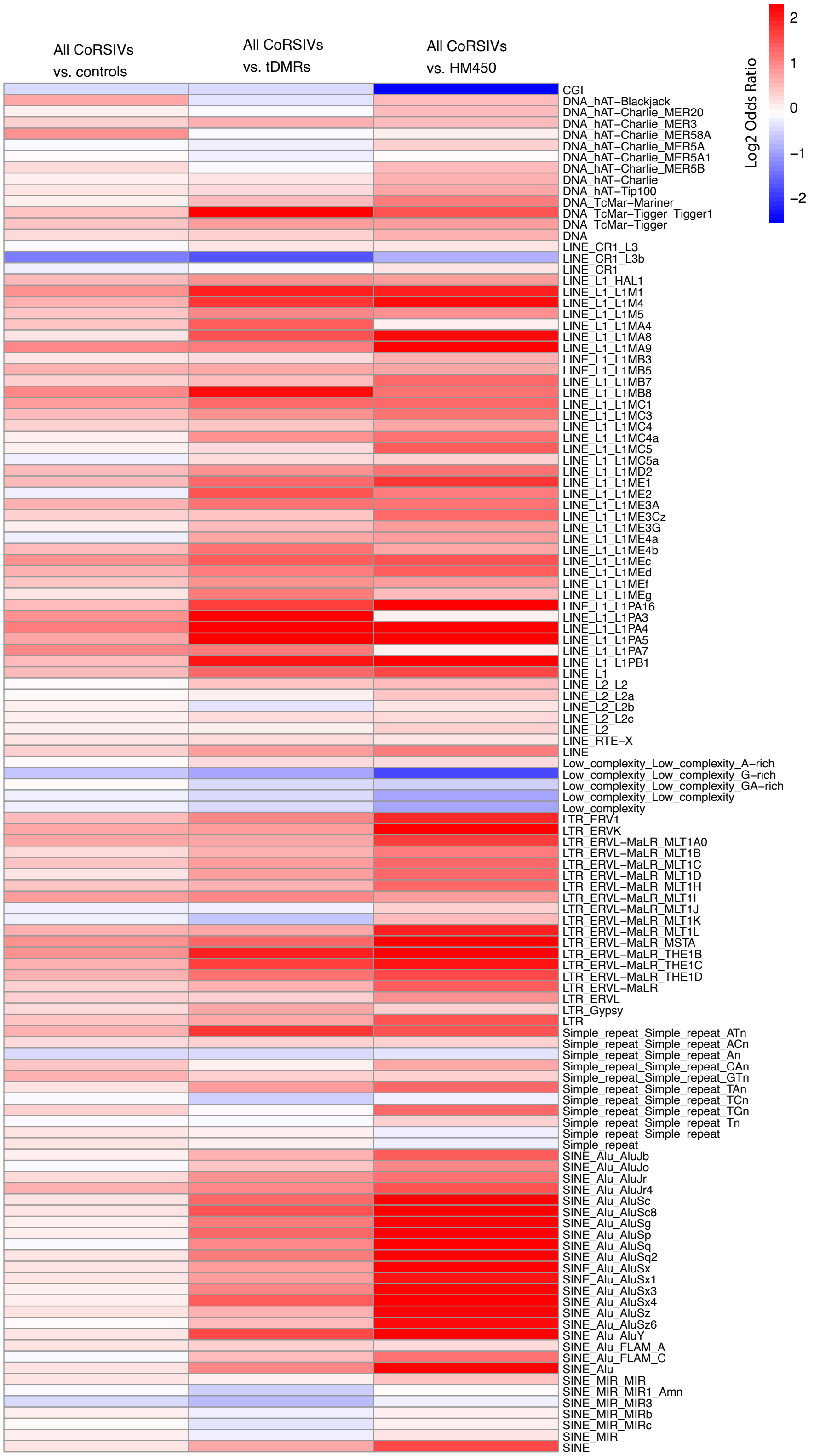

Fig. S19. Overlap of transposable elements over all CoRSIV regions compared to controls, tDMRs and HM450

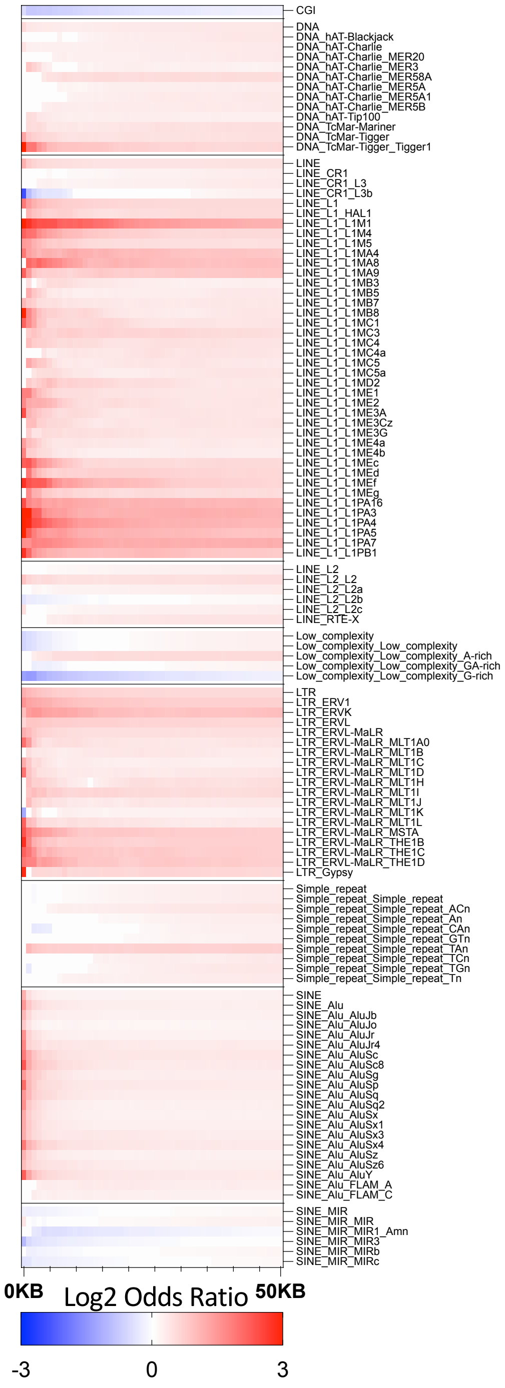

Fig. S20. Transposable element enrichment for Genic CoRSIVs vs. tDMRs

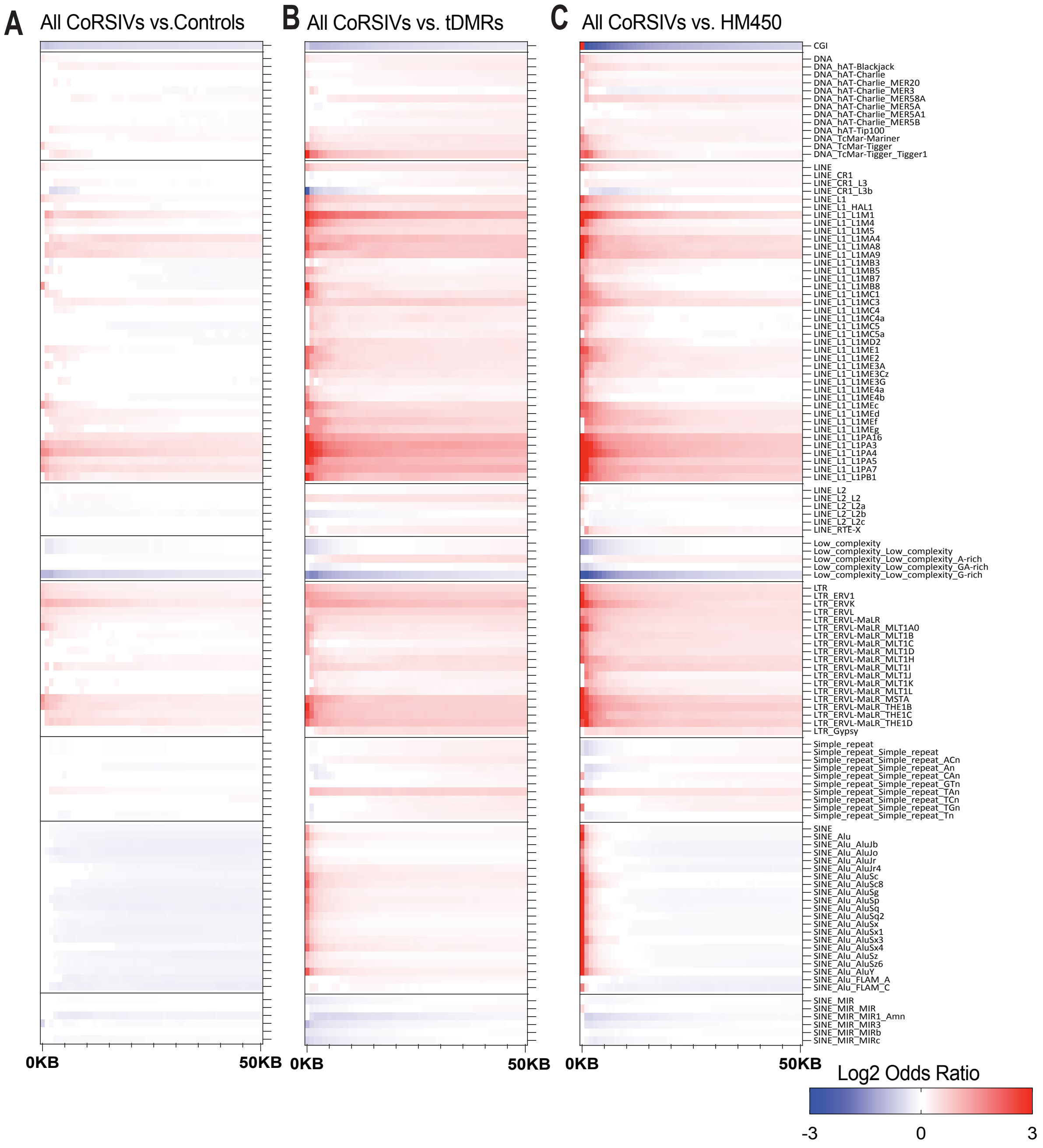

Fig. S21. Transposable element enrichment for (A) All CoRSIVs vs. controls, (B) All CoRSIVs vs. tDMRs, and (C) All CoRSIVs vs. HM450

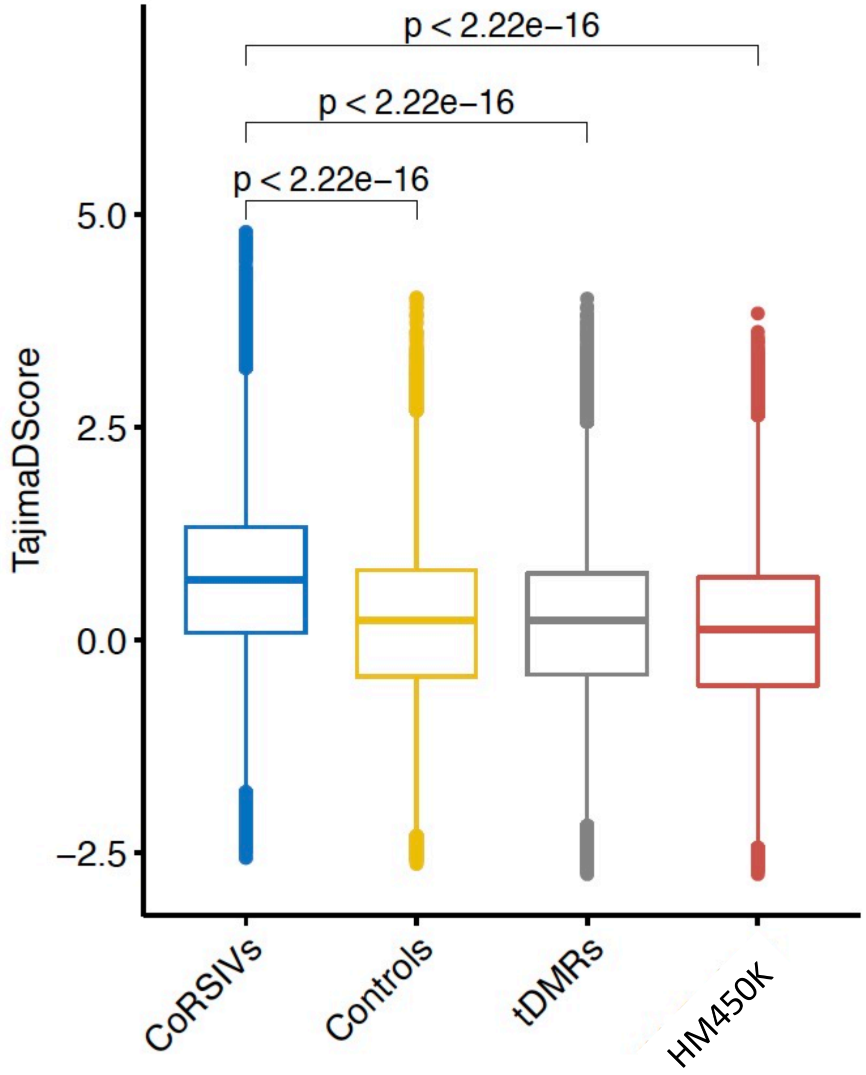

Fig. S22. Tajima's D Score distributions for CoRSIVs, Controls, tDMRs, and HM450 probes. Tajima's D is higher in CoRSIVs than in the other regions, providing evidence of evolutionary selection.

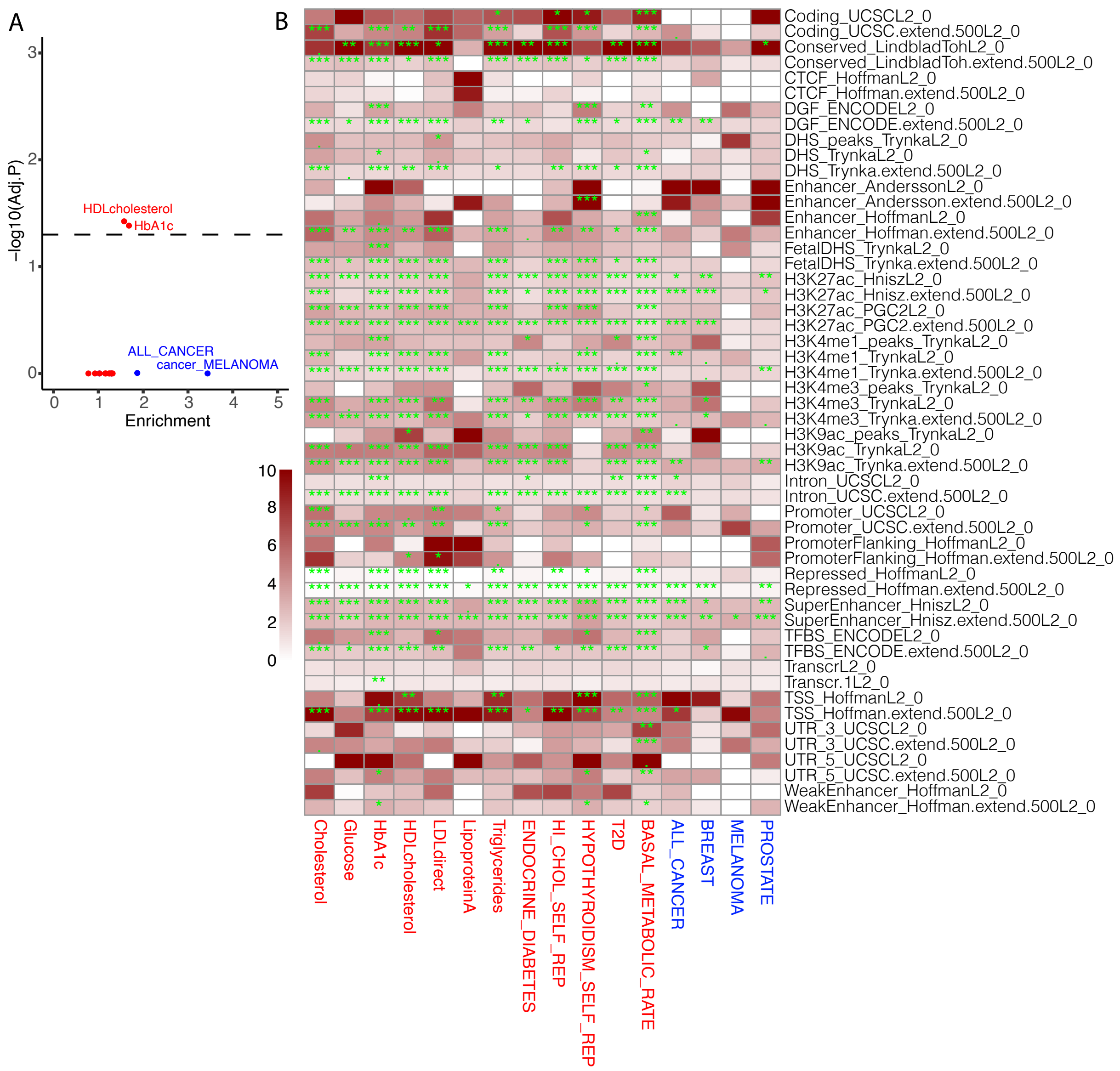

Fig. S23. LDSC heritability enrichment score for SNVs in CoRSIV +/- 20kb , when 53 baseline features are included in the model. (A) LDSC enrichment score vs. Bonferroni adjusted p-value in -log10 scale for 12 metabolic traits and 4 cancer outcomes when CoRSIV +/- 20kb region and full 'baseline' features including 53 sequence and epigenomic features are included in the models. **(B)** LDSC Enrichment and Bonferroni adjusted p-value (green color) for 53 baseline sequence and epigenomic features when used in the models with CoRSIV +/- 20kb SNVs. 0 - 0.001 = '***', 0.001 - 0.01 = '**', 0.01 - 0.05 = '*', 0.05 - 0.1 = '.', 0.1 - 1.0 = ' ' .

References:

1. M. J. Silver *et al.*, Independent genomewide screens identify the tumor suppressor VTRNA2-1 as a human epiallele responsive to periconceptional environment. *Genome Biol* **16**, 118 (2015).

2. T. E. Van Baak *et al.*, Epigenetic supersimilarity of monozygotic twin pairs. *Genome Biol* **19**, 2 (2018).
3. D. Monk *et al.*, Recommendations for a nomenclature system for reporting methylation aberrations in imprinted domains. *Epigenetics* **13**, 117-121 (2018).
4. E. A. Houseman *et al.*, DNA methylation arrays as surrogate measures of cell mixture distribution. *BMC Bioinformatics* **13**, 86 (2012).
5. A. E. Jaffe, R. A. Irizarry, Accounting for cellular heterogeneity is critical in epigenome-wide association studies. *Genome Biol* **15**, R31 (2014).
6. C. J. Gunasekara *et al.*, A genomic atlas of systemic interindividual epigenetic variation in humans. *Genome Biol* **20**, 105 (2019).
7. C. H. Wei, A. Allot, R. Leaman, Z. Lu, PubTator central: automated concept annotation for biomedical full text articles. *Nucleic acids research* **47**, W587-W593 (2019).
8. A. R. Quinlan, I. M. Hall, BEDTools: a flexible suite of utilities for comparing genomic features. *Bioinformatics* **26**, 841-842 (2010).
9. F. Krueger, S. R. Andrews, Bismark: a flexible aligner and methylation caller for Bisulfite-Seq applications. *Bioinformatics* **27**, 1571-1572 (2011).
10. H. Thorvaldsdottir, J. T. Robinson, J. P. Mesirov, Integrative Genomics Viewer (IGV): high-performance genomics data visualization and exploration. *Briefings in bioinformatics* **14**, 178-192 (2013).
11. R. Luijk, J. J. Goeman, E. P. Slagboom, B. T. Heijmans, E. W. van Zwet, An alternative approach to multiple testing for methylation QTL mapping reduces the proportion of falsely identified CpGs. *Bioinformatics* **31**, 340-345 (2015).
12. P. Du *et al.*, Comparison of Beta-value and M-value methods for quantifying methylation levels by microarray analysis. *BMC bioinformatics* **11**, 587 (2010).
13. A. A. Shabalin, Matrix eQTL: ultra fast eQTL analysis via large matrix operations. *Bioinformatics* **28**, 1353-1358 (2012).
14. C. C. Chang *et al.*, Second-generation PLINK: rising to the challenge of larger and richer datasets. *Gigascience* **4**, 7 (2015).
15. S. B. Gabriel *et al.*, The structure of haplotype blocks in the human genome. *Science* **296**, 2225-2229 (2002).
16. C. Zhang *et al.*, European genetic ancestry associated with risk of childhood ependymoma. *Neuro Oncol* **22**, 1637-1646 (2020).
17. J. L. Min *et al.*, Genomic and phenomic insights from an atlas of genetic effects on DNA methylation. *medRxiv*, 2020.2009.2001.20180406 (2020).
18. F. Tajima, Statistical method for testing the neutral mutation hypothesis by DNA polymorphism. *Genetics* **123**, 585-595 (1989).
19. M. Pybus *et al.*, 1000 Genomes Selection Browser 1.0: a genome browser dedicated to signatures of natural selection in modern humans. *Nucleic acids research* **42**, D903-909 (2014).
20. M. J. Bonder *et al.*, Disease variants alter transcription factor levels and methylation of their binding sites. *Nat Genet* **49**, 131-138 (2017).
21. H. K. Finucane *et al.*, Partitioning heritability by functional annotation using genome-wide association summary statistics. *Nature genetics* **47**, 1228-1235 (2015).
